# A Computational Model for Assessing the Population Health Impact of Introducing a Modified Risk Claim on an Existing Smokeless Tobacco Product

**DOI:** 10.3390/ijerph16071264

**Published:** 2019-04-09

**Authors:** Raheema S. Muhammad-Kah, Yezdi B. Pithawalla, Edward L. Boone, Lai Wei, Michael A. Jones, Ryan A. Black, Thomas M. Bryan, Mohamadi A. Sarkar

**Affiliations:** 1 Regulatory Affairs, Center for Research & Technology, Altria Client Services LLC, 601 East Jackson Street, Richmond, VA 23219, USA; Yezdi.B.Pithawalla@altria.com (Y.B.P.); Lai.Wei@altria.com (L.W.); Michael.A.Jones@altria.com (M.A.J.); Ryan.A.Black@altria.com (R.A.B.); Thomas.M.Bryan@altria.com (T.M.B.); Mohamadi.A.Sarkar@altria.com (M.A.S.); 2 Department of Statistical Sciences & Operations Research, Virginia Commonwealth University, Richmond, VA 23284, USA; elboone@vcu.edu

**Keywords:** population modeling, tobacco harm reduction, modified risk tobacco products, smokeless tobacco, modified risk tobacco product application, population level impact

## Abstract

Computational models are valuable tools for predicting the population effects prior to Food and Drug Administration (FDA) authorization of a modified risk claim on a tobacco product. We have developed and validated a population model using best modeling practices. Our model consists of a Markov compartmental model based on cohorts starting at a defined age and followed up to a specific age accounting for 29 tobacco-use states based on a cohort members transition pathway. The Markov model is coupled with statistical mortality models and excess relative risk ratio estimates to determine survival probabilities from use of smokeless tobacco. Our model estimates the difference in premature deaths prevented by comparing Base Case (“world-as-is”) and Modified Case (the most likely outcome given that a modified risk claim is authorized) scenarios. Nationally representative transition probabilities were used for the Base Case. Probabilities of key transitions for the Modified Case were estimated based on a behavioral intentions study in users and nonusers. Our model predicts an estimated 93,000 premature deaths would be avoided over a 60-year period upon authorization of a modified risk claim. Our sensitivity analyses using various reasonable ranges of input parameters do not indicate any scenario under which the net benefit could be offset entirely.

## 1. Introduction

According to the Surgeon General, cigarette smoking is the single greatest cause of avoidable morbidity and mortality in the United States (U.S.) [1]. In the U.S., cigarette smoking causes about one in every five deaths annually and the life expectancy of cigarette smokers is about 10 years shorter than non-cigarette smokers [2]. According to the Centers for Disease Control and Prevention, “smoking is the primary causal factor for at least 30% of all cancer deaths, for nearly 80% of deaths from chronic obstructive pulmonary disease, and for early cardiovascular disease and deaths” [2]. The American Cancer Society further states that 80% of all lung cancer deaths are caused by smoking [3]. Smoking cessation is the most effective means of reducing the risk of smoking-related disease, including lung cancer. However, for those who are unable or unwilling to quit smoking, completely switching from smoking cigarettes to using a less hazardous non-combustible tobacco product might reduce the risk of smoking-related diseases.

Moist smokeless tobacco (MST) products are non-combustible tobacco products that have been available in the U.S. for decades. The U.S. Surgeon General and other public health authorities have determined that MST products are addictive and are not risk-free as noted in federally mandated warnings. However, a vast body of epidemiological evidence exists demonstrating that MST products are substantially less hazardous than cigarettes [4,5].

We have submitted a modified risk tobacco product application (MRTPA) to the U.S. Food and Drug Administration (FDA) on one of the MST products manufactured by U.S. Smokeless Tobacco Company. The FDA requires that an MRTPA demonstrates that authorization of a modified risk claim for a tobacco product will benefit the health of the population as a whole, taking into account both users and never-users of tobacco products [6,7].

In recent years, several mathematical models have assisted in predicting the potential public health impact of change in use of tobacco products with varying levels of inherent risk. Bachand and Sulsky [8] and Bachand et al. [9] used cohort-based compartmental models to assess the introduction of an MRTP on all-cause mortality. Apelberg et al. [10] used the dynamic systems population model described by Vugrin et al. [11] to quantify the prevalence of tobacco use and potential public health effects (i.e., tobacco-related mortality, and life years gained) of enacting U.S. regulation intended to make cigarettes minimally addictive by reducing their nicotine content levels. Other researchers [12,13] have used a Markov model combined with a negative exponential mortality model to estimate the effect of introducing a reduced risk tobacco product on hypothetical European and U.S. population samples of 10,000 individuals, respectively. Hill and Camacho [14] used a system-dynamics-based compartmental stock and flow model to assess the potential health impact of launching a new MRTP into the marketplace. Levy et al. [15] developed a status quo scenario that projected smoking rates and health outcomes in the absence of e-cigarette use and compared it with substitution models, where cigarette smoking is largely replaced by e-cigarette use over a specific time. Poland et al. [16] developed a statistical model based on public data to explore the effect of introducing an MRTP that ultimately resulted in reduced conventional cigarette smoking on population mortality.

At Altria Client Services LLC (ALCS), we have developed a computational model (henceforth referred to as the ALCS Cohort Model) using similar principles as those described in the literature to predict population harm from tobacco products. This model uses best practices described by the Modeling Good Research Practices Taskforce, a joint task force developed by the International Society for Pharmacoeconomics and Outcomes Research (ISPOR) and the Society for Medical Decision Making (SMDM), for the development of mathematical models for health care and public health decision-making [17]. We describe the development, validation, and application of this model to determine the population impact of authorization of a modified risk claim on an existing MST product.

## 2. Methods and Analysis

### 2.1. Model

The ALCS Cohort Model follows the survival of a hypothetical cohort population as it ages over a specific timeframe. The cohort population in the simulation starts at the same age and consists of never-users of tobacco products.

Our computational model is based on the Markov compartmental model approach, which uses distinct states and transition probabilities to account for the various possible progression within the model. Age-specific transition probabilities are used to propagate the cohort through the various states of tobacco use and nonuse across time. The Markov model is coupled with mortality models that use excess relative risk (ERR) ratios to determine survival probabilities of the cohort at the end of a consecutive five-year interval. The model was employed within a single cohort format, in which a fixed cohort population was followed from a particular age to end of life. We also extended the single-cohort model to a time-staggered, multi-cohort model to estimate the net benefit representative of the population. Our single cohort model structure is similar to one adopted by Bachand and Sulsky [8].

The ALCS Cohort Model is implemented using a Bayesian framework with parameter estimates that use Markov Chain Monte Carlo techniques to account for uncertainties associated with input parameters. We implemented the ALCS Cohort Model using the MCMCpack (i.e., Bayesian approach via the Markov Chain Monte Carlo procedure) package version 1.4-4 and msm (i.e., multi-state modeling) package Version 1.6.6 in R statistical programming, Version 3.5.1(R Core Team, Vienna, Austria) [18].

### 2.2. Framework for Estimating the Impact of a Market Authorization of a Modified Risk Claim on an Existing Moist Smokeless Tobacco (MST) Product

**Figure 1:**
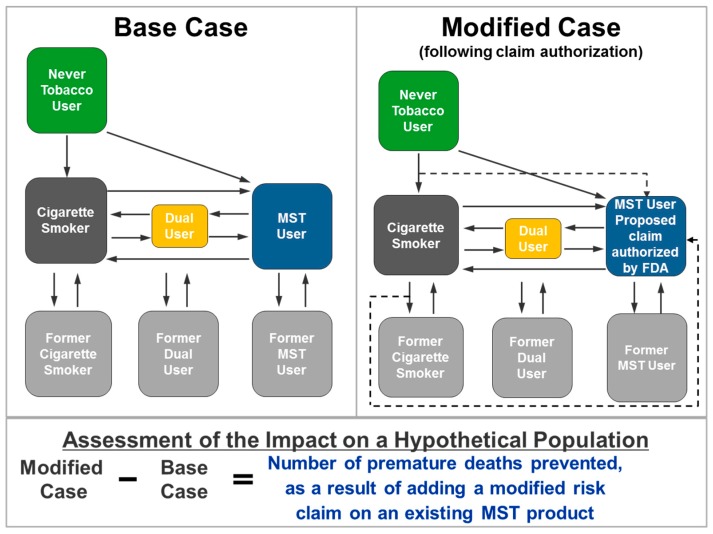
Framework for determining the overall population health impact of a modified risk claim on an existing moist smokeless tobacco (MST) product.

The framework presented in this paper is used to estimate the overall population health impact from FDA authorization of a modified risk claim on an existing MST product by comparing the survival of hypothetical populations in the two scenarios (i.e., number of premature deaths prevented) depicted in Figure 1. More details regarding the transition pathways are shown in Supplementary File 1 (Figure S1.1).

#### 2.2.1. Base Case Scenario

The Base Case or status quo scenario takes into consideration the tobacco use behaviors in a U.S. native-born male population that includes various transitions for both cigarette smokers and MST users. We consider only males for this simulation because > 90% of smokeless tobacco (ST) product users in the U.S are males [19]. Our Base Case scenario is unique in that it captures the tobacco use behavior of the two most predominant forms of tobacco products (cigarette smoking and MST use), compared to several published models that focus on estimating the effect of introducing a new tobacco product into the market and consider a status quo scenario of cigarette use only [8,9,11,12,13,14,15,16]. Transition probabilities were attainable for the Base Case from peer-reviewed literature given the long history of usage of cigarettes and MST in the U.S. native-born male population.

#### 2.2.2. Modified Case Scenario

The Modified Case scenario reflects a future state in which we assess potential changes to the Base Case due to market authorization of a modified risk claim on an existing MST product.

We first estimated the impact at the MST category level by determining the number of premature deaths prevented by following a single cohort of 1,000,000 males, beginning the simulation at age 13 years assuming they have never used any tobacco products. Next, we estimated the number of premature deaths prevented in a representative, U.S. native-born male population by extending the single-cohort model to a time-staggered, multiple-cohort model. Since the multiple-cohort model is based on U.S. Census projections, these estimates are expected to be representative of the population.

### 2.3. Single-Cohort Model

#### 2.3.1. Transition Sub-Model

This sub-model uses a Markov chain approach to track the transitions between various states in the model. At any time, an individual (cohort member) may reside in only one usage state with the probability of moving to a different use state at the next time point. To estimate whether an individual (cohort member) belongs in a specific usage state, we considered age-specific transition paths between states. We employed 29 states and specified 30 input parameters that were used to estimate the transition probabilities, which are used to propagate the cohort through the various states across time. An illustration depicting the states and transitions are shown in Supplementary File 1 (Figure S1.1). We introduced uncertainty in initiation and cessation rates by drawing these rates from a truncated normal distribution ranging from 0–100% with the mean parameter being the rate of initiation or cessation at each cohort age category and a standard deviation of 2%.

(a) Transition Probabilities for the Base Case Scenario

The Base Case is composed of nonusers of tobacco products, cigarette smokers, and MST users, reflecting the status quo. The transition probabilities were estimated from a systematic review of published literature on transitions between smokeless tobacco use and cigarette smoking in the U.S. [20]. We used only those studies from the systematic review in which the underlying populations reported in the studies were most generalizable to the U.S. population; male-specific transition rates were reported, and the transition time periods were most similar to those used in our modeling analyses [21,22,23]. Estimation of transition probabilities from the relevant studies reviewed within Tam et al. [20], used in the Base Case scenario, are discussed in Supplementary File 2.

(b) Transition Probabilities for the Modified Case Scenario

The Modified Case scenario reflects a future state in which the transitions within the population potentially change due to authorization of a modified risk claim on an existing MST product. The potential percent change by which Base Case transition probabilities would change in the Modified Case scenario was obtained from a large study (Claim Comprehension and Intentions Study, CCI Study), in which we measured changes in behavioral intentions in different groups of adult tobacco users and nonusers.

The CCI Study is a quasi-randomized online study involving 5871 participants. The study population was composed of a non-probability sample of qualified adults (legal age to purchase tobacco products or older), self-reported tobacco product users and nonusers. Participants were recruited to match the U.S. population on major demographic variables using quotas from the Population Assessment of Tobacco and Health (PATH) Study [24]. The study protocol and survey questionnaire were reviewed by an Institutional Review Board (Chesapeake IRB, Columbia MD). The IRB determined that this was exempt from IRB oversight based on the Department of Health and Human Services regulations found at 45 CFR 46.101(b)(2). Since the study was exempt from IRB review there was no IRB approval number. All participants gave their informed consent electronically before participating in the study.

One of the primary outcomes of the study was to assess changes in behavioral intentions between the Test condition (measured before and after exposure to an advertisement of the MST product with the modified risk claim) and the Control condition (measured before and after exposure to an advertisement of the MST product without the modified risk claim). The behavioral intention metrics used in the CCI study were developed in accordance with FDA guidance and standards in psychometrics [25,26].

We note that actual behavior use patterns observed under “real-world” conditions may differ from the behavioral intentions measured in the CCI study. For example, research has generally found a moderate relationship between behavioral intentions and actual behaviors [27]. In an effort to translate behavioral intentions to actual behavior, we defined likelihood of behavior (i.e., switch, use, dual use) for each participant based on an average rating on the behavioral intention items (i.e., Response Option Scale: 1 = Strongly Disagree, 2 = Disagree, 3 = Somewhat Disagree, 4 = Somewhat Agree, 5 = Agree, 6 = Strongly Agree) along with intention to purchase the product (no/yes). Participants who had both high behavioral intentions (defined as an average score corresponding to Somewhat Agree or higher) and endorsed intentions to purchase the product were coded as likely to engage in behavior. These criteria were developed in an effort to balance sensitivity and specificity while taking into account over-inflation of behavior when using self-report intention data [27]. The pre-post change in the proportion of participants who were considered likely to engage in the behavior in the test condition (i.e., those exposed to the claim in the context of an ad) was compared to the pre-post change in the control condition (i.e., those only exposed to an ad). The relative difference between conditions on the pre-post change in proportions was used to estimate the impact of exposure to the claim (above and beyond a typical ad) on Base Case transition rates. Specifically, we applied the estimated relative percent difference estimated from the CCI Study to the Base Case transition probabilities to generate the corresponding rates for key transitions for the Modified Case scenario. For example, applying the relative percentage change of 21% for current cigarette smokers switching completely to the MST product with a claim results in a transition rate of 1.7% for the Modified Case ((1.4% × 0.21) + 1.4%), given that the Base Case transition is reported as 1.4% in published literature.

Table 1 summarizes the change in proportion of respondents between the Test and Control groups who demonstrated likelihood to engage in behavior, when they were exposed to advertising material with and without the modified risk claim. A relative percentage change estimated as a negative value suggests that exposure to the proposed modified risk claim reduces the likelihood of switching to the MST product compared to the control. Details regarding estimation of input parameters are provided in Supplementary File 2.

Two additional transitions that are new behaviors as a result of the authorized claim—“would-be smoker” and “would-be smoking quitter”—could not be studied in the CCI Study because of the impracticality of assessing these transitions in a pre-market survey setting.

Would-be smokers are never tobacco users, who would have otherwise started smoking cigarettes, but instead, start using ST, creating a pathway for intercepting smokers. Would-be smoking quitters are cigarette smokers who would have otherwise quit smoking cigarettes, but instead, switch to ST. Transition rates for “would-be smoker” and “would-be smoking quitter” were assumed to be 1% and 5%, respectively. A sensitivity analysis using a wide range (0–100%) was conducted given the theoretical nature of these two transitions.

#### 2.3.2. Mortality Sub-Models

The transition sub-model is coupled with Poisson regression models used to estimate the number of survivors of each age category at the end of the simulation. A Kaiser–Permanente (KP) Medical Care Program Cohort study provided person-years and number of deaths due to all causes for never-users of tobacco, former cigarette smokers, and current cigarette smokers by age group, gender, duration of smoking, and duration of quitting cigarettes [28]. We further adjusted the KP mortality data to be more representative of the U.S. population. Details of the derivation of the mortality data are shown in Supplementary File 2.

The Poisson model included the following predictors: age (AGE), years smoked (YSM), and years since quitting smoking (YQSM). A second-order term AGE^2^, and interaction terms (AGE × YSM and AGE × YQSM) were also included in the models to improve the overall fit. Three separate models were created for never-users of tobacco, cigarette smokers, and former cigarette smokers, which were fitted to their corresponding datasets using the natural log link:

(1) $$MR_{i}\sim Poisson\left(\lambda_{i}\right)$$

where MR is the mortality rate corresponding to each 100,000 people, λ is the mean mortality rate, and *i* represents members within a specific user group (never users, cigarette smokers, and former smokers).

(2) $$\ln\left(\lambda_{i}\right)=\beta_{0}+\beta_{1}AGE_{i}+\beta_{2}AGE_{i}^{2}\;(\mathrm{Never}\;\mathrm{Users}\;\mathrm{of}\;\mathrm{Tobacco})$$

(3) $$\ln\left(\lambda_{i}\right)=\beta_{0}+\beta_{1}AGE_{i}+\beta_{2}AGE_{i}^{2}+\beta_{3}YSM_{i}+\beta_{4}YSM_{i}\times AGE_{i}\;(\mathrm{Current}\;\mathrm{Cigarette}\;\mathrm{Smokers})$$

(4) $$\ln\left(\lambda_{i}\right)=\beta_{0}+\beta_{1}AGE_{i}+\beta_{2}AGE_{i}^{2}+\beta_{3}YSM_{i}+\beta_{4}YQSM_{i}+\beta_{5}YSM_{i}\times AGE_{i}+\beta_{6}YSQM_{i}\times AGE_{i}\;(\mathrm{Former}\;\mathrm{Cigarette}\;\mathrm{Smokers})$$

To estimate mortality rates of MST users (i.e., both current and former users), we employed the concept of using an ERR ratio [29], which quantifies the differential risk posed by using MST compared to smoking cigarettes. The ERR ratio between exclusively using the MST product versus smoking cigarettes is derived as shown in Equation (5):

(5) $$\mathrm{ERR}\;\mathrm{Ratio}\;\left(\mathrm{MST}\;\mathrm{vs.}\;\mathrm{cigarette}\;\mathrm{smoker}\right)=\frac{\left[\mathrm{Relative}\;\mathrm{Risk}\;\left(\mathrm{Using}\;\mathrm{MST}\;\mathrm{to}\;\mathrm{baseline}\;\mathrm{never}\;\mathrm{smoker}\right)-1\right]}{\left[\mathrm{Relative}\;\mathrm{Risk}\;\left(\mathrm{Cigarette}\;\mathrm{smoking}\;\mathrm{to}\;\mathrm{baseline}\;\mathrm{never}\;\mathrm{smoker}\right)-1\right]}$$

On a reference scale of 0–1, the baseline ERR ratio is set to 0 for never-tobacco users and 1 for current cigarette smokers. We estimated relative risks from the mortality hazard ratios (HR) for ST use and for cigarette smoking. The relative risk estimates were derived from our analyses of two large, nationally representative linked mortality datasets. We analyzed data from the National Health Interview Survey (NHIS) and the National Longitudinal Mortality Survey (NLMS) that have been linked with mortality information from the National Death Index (NDI) by the National Center for Health Statistics (NCHS). We estimated the mortality HR using a Cox Proportional Hazards Model [30]. The ERR ratio used in the model for comparing the risks of ST users relative to the risks of cigarette smokers based on the all-cause mortality HR estimates were derived from using the publically available NHIS datasets. The ERR ratios of current ST users compared with current cigarette smokers and former ST users compared with former cigarette smokers were estimated to be 0.09 and 0.04, respectively. The interpretation of the 0.09 value is that exclusive smokeless tobacco use has 90% less excess risk than exclusive cigarette smoking. In addition, we assigned the mortality risk of dual use (concurrent cigarette and ST) to be the same as the mortality risk of exclusive cigarette use based on the published literature [31,32]. Using ERR ratios and mortality risk related to never tobacco use, current cigarette use, and former cigarette use, we were able to derive the accumulated risks associated with spending time in each state based on a cohort-members-specific pathway. Mortality rates for a specific pathway are the product of the users’ risk based on their switching/usage patterns. For example, the mortality risks for an individual who was a cigarette smoker but who switched to exclusive MST use is derived from the product of four components: (1) Background risk, which is risk of never tobacco use; (2) the relative risk due to smoking for the period of time the person was a smoker; (3) the relative risk due to current MST use; and (4) the relative risk associated with the period of time the person has quit smoking and was a former smoker. Table S1.1 (Supplementary File 1) provides details regarding how we derived the accumulated risks associated with spending time in each state based on a cohort member’s specific pathway.

**Figure 2:**
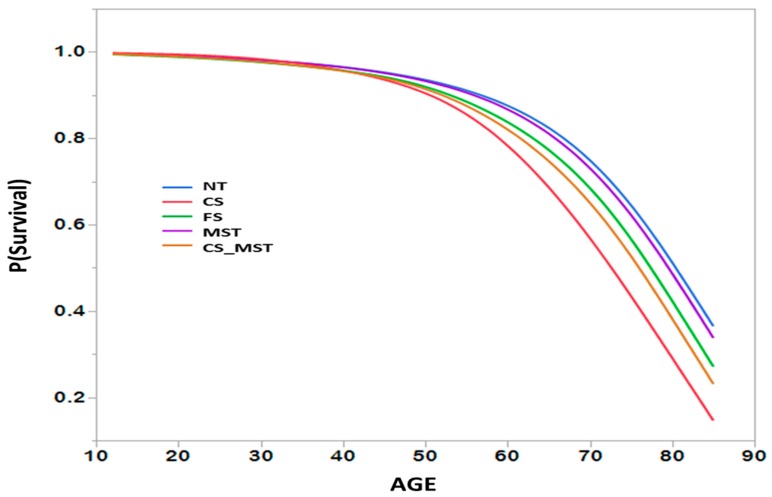
Example of Cumulative Survival Curves. Note: NT-Never tobacco user; CS-Current cigarette smoker; FS-Former cigarette smoker; MST-Exclusive Moist smokeless tobacco user; CS_MST-Cigarette smoker who quit smoking and switched to exclusive MST Use; ERR_(MST|CS)_-excess relative risk ratio = 0.09.

Based on the mortality risks formulas, gender and age-specific survival curves can be created for different groups within the cohort population, where *p* (Survival) represents the probability of being alive at a particular age. Figure 2 below is an example of survival curves for males to demonstrate the difference in survival probabilities for individuals within the cohort being in a different tobacco use state (Never tobacco user, current cigarette smoker, former cigarette smoker, exclusive MST user, and cigarette smokers who switch to exclusive MST use). The estimated survival probabilities are in agreement with expected trends. At higher ages, the probability of survival is lowest for current cigarette smokers, followed by cigarette smokers who switch to MST use and then cigarette smokers who quit all tobacco use.

Additional details on estimations of the HR and ERR ratios are presented in Supplementary File 2. Also, the model pseudo code, a simplified version of the single cohort code, is presented in Section 1.4 of Supplementary File 1.

### 2.4. Multiple-Cohort Model

Single-cohort models are designed to take a specific homogeneous group and track them as they age over time, using defined transition probabilities and mortality rates. In order to make inferences on a more heterogeneous population, we employed a time-staggered, multiple-cohort approach that follows multiple individual birth cohorts, each being different in size beginning with their birth year and corresponding population size of U.S. native-born males based on the U.S. Census estimates [33]. Additional details and an illustration explaining how the multiple-cohort approach is applied are presented in Section 1.2 of Supplementary File 1.

For each cohort, corresponding age- and gender-specific transition rates and mortality models were applied, which produces a reduction in the cohort size as the cohort moves through time.

#### 2.4.1. Transition and Mortality Probabilities

In the Base Case scenario, for each cohort, we incorporated initiation and cessation rates for the single cohort model from Anderson et al. [34]. The supplemental data in Anderson et al. [34] reported initiation and cessation rates by age for five-year birth cohorts of males born between 1910–1980 on a yearly basis. For initiation and cessation rates of males born after 1980, and for transition probabilities involving exclusive MST use and dual use, we used the rates estimated by Tam et al. [20] and kept them constant over the entire period. The initial cohort size based on their birth cohort year for U.S. native-born males was obtained from U.S. Census data [33,35]. Similar to the approach used in the single-cohort model, we applied the transition rates based on the proportion of individuals likely to engage in behavior, as defined previously, for the key transitions (described in Table 1) to the Base Case to generate the corresponding transition rates for the Modified Case scenario.

#### 2.4.2. Model Assumptions

At each step of the model development and analysis, efforts were made to use both reasonable assumptions and model parameter estimates. A key assumption is that the KP dataset used to create the mortality models is representative of the general population. However, the study participants had health insurance, short follow-up periods, and their age-specific mortality rates were lower than those for the U.S. population [28]. We therefore adjusted the dataset by assigning weights that reflect mortality rates observed in the U.S. population in the year 2000 using U.S. Vital Statistics. We note that, apart from the KP dataset, very few publically available data of this nature exist in the literature, especially with the attributes of “number of years smoked” and “years since cessation” and their impact on gender-specific all-cause mortality. A similar approach has been published in the peer-reviewed literature by Bachand and Sulsky [8].

A possible limitation of our mortality model is that it does not currently take into account cigarette per day. We make the assumption that daily and non- daily cigarette use has the same mortality risk. This assumption is in line with published literature that indicates while non-daily smokers may have lower mortality risks it is still substantial [36]. Additionally, although numerous transition states can possibly occur, we assume that the 29 states are sufficiently specified to account for all reasonable paths of use over time. These 29 states in the model allow for switching between products, in addition to cessation of product use.

We also assume that the changes to transition rates from the CCI Study will remain approximately constant over the modeling time period. Similarly, we assume that the product-specific initiation, cessation, and other transition rates do not change over the modeling time period and that age- and product use state-specific mortality rates remain constant over the modeling time period.

Although poly-tobacco (three or more) use has been observed among U.S. adult tobacco users [24], however in order to limit the transitions to a manageable number we do not include transitions to other tobacco products (e.g., cigars, e-vapor products). In addition there is insufficient data to estimate numerous possible transition probabilities between different tobacco product use states. In our model we assume that much of the poly-tobacco use is not regular daily use but occasional non-daily use. Our analysis of PATH Wave 1 showed that 0.3% of adults used a combination of cigarettes, e-cigarettes and smokeless tobacco of which e-cigarette and smokeless tobacco use was “some day”. Also a peer-reviewed analysis of PATH Wave 1 found that multiple-product use (i.e., current use of three products) accounted for around 1% of adult poly-tobacco use [37].

## 3. Results and Discussion

### 3.1. Model Validation

#### 3.1.1. Validation of Single-Cohort Approach

To validate our Base Case scenario (i.e., world as-is today with cigarettes and MST), we compared our model predictions against the number of survivors estimated using mortality data reported in the 2006 U.S. Life Table from the National Vital Statistics Report [38]. In order to predict mortality in the year 2006, we needed to use age- and gender-specific rates (e.g., Initiation and Cessation rates) from the year 1980 to provide a suitable introduction period. This captures the best time frame in which smoking-related mortality would impact the cohort [39,40]. We calculated the predicted means and standard deviations using 10,000 samples from the posterior predictive distribution. The percent difference between the predicted mean and the reported mean for each age group yielded a relatively low percent difference for all age groups, ranging from 0.28–3.24% the results are shown in Supplementary File 3. As expected in terms of all-cause mortality, we observe a closer agreement between the two estimates in the older ages when tobacco-related mortality is more prevalent.

#### 3.1.2. Validation of Multiple-Cohort Approach

We validated the multiple-cohort approach by comparing the total population (0–104 years of age) generated by the model to that reported by the 2015 U.S. Census [33] in the Base Case scenario. We built a population of U.S. native-born males living in the year 2015 with ages ranging from 0–104 years. Since our cohort model operates in five-year intervals, we initiated the first cohort of ages 0–4 years in the time period 1910–1914 and modeled the survival of that single cohort over a period of 104 years up to the year 2015. Comparisons showed that our projected population estimates are within 2.27% of population estimated by the U.S. census, for the 2015 U.S. native-born male population (i.e., 140,297,321 model estimate versus 137,187,000) [35]. More details can be found Supplementary File 3.

We further validated the multiple-cohort approach by comparing the model population estimate projections with U.S. native-born male population projections from the U.S. Census Bureau at each five year time interval from the 2020–2060 timeframe. The comparison revealed relatively small differences between the model and Census Bureau projections at any of the time points, ranging from 0.11–5.21%.

Comparisons between results from our model and the U.S. Census data show that the multiple-cohort model with a time-staggered structure can successfully model the U.S. native-born male population.

### 3.2. Estimating the Impact of Authorization to Market Our Candidate MST With a Modified Risk Claim

#### 3.2.1. Estimating the Number of Premature Deaths Prevented between Base and Modified Case Scenarios Using the Single-Cohort Approach

In the single-cohort approach, we follow the survival of a hypothetical cohort of 1,000,000 males, starting at age 13 years, as they age under the defined Base and Modified Case scenarios. Our results are presented as differences in the number of premature deaths prevented between these two scenarios. The results are presented as point estimates with posterior credible intervals to address model input parameter uncertainty. Table 2 shows the results of the modeling outcomes of the Base Case and Modified Case scenarios.

The impact of our most likely changes in tobacco use patterns as a result of authorization of a modified risk claim resulted in 1120 premature deaths prevented with 32,856 additional years of expected life, for a cohort consisting of 1,000,000 males in the U.S. followed from age 13–73 years. Table 3 shows the additional years of expected life derived by using standard Life Table measurements [41]. A key driver of the number of premature deaths prevented in the Modified Case scenario is the years of life gained by being in a lower relative risk state (i.e., switching from cigarette smoking to use of the lower risk candidate product) compared with being in a higher relative risk state (i.e., continue smoking cigarettes). Although not shown here, if we followed the two cohorts beyond age 73 years, we would see a declining trend of differences between the Base Case and Modified Case. This trend is likely due to a decline in mortality HRs associated with smoking in males at older ages, as reported by Rostron [42]. Since the outcome of interest for the ALCS cohort model is difference in all-cause mortality between our Base Case and Modified Case Scenarios, we focused on the age group where the greatest cumulative difference in mortality would likely be observed (i.e., the 73-year age group).

#### 3.2.2. Estimating Differences in Number of Survivors between Base and Modified Case Scenarios Using the Multiple-Cohort Approach

Table 4 shows the results of the Modified and Base Case scenarios across age groups and the difference in the number of premature deaths prevented between the two scenarios for males alive between ages 0–84 years. The difference increases rapidly from ages 40–74 years, before beginning to decline slightly. This trend also aligns with the observation and explanation by Rostron [42], who showed that mortality ratios associated with smoking in males increase with age from 45–74 years, before slightly declining at older ages [42]. Under the defined modeling scenarios, we predict that authorization of the modified risk claim will likely prevent 93,323 premature deaths at the end of a 60-year follow-up period compared with the status quo.

To understand the contribution of each of the key transitions, we applied the Modified Case transition probabilities to one transition at a time, while keeping all other transitions at the same rates as the Base Case scenario.

**Figure 3:**
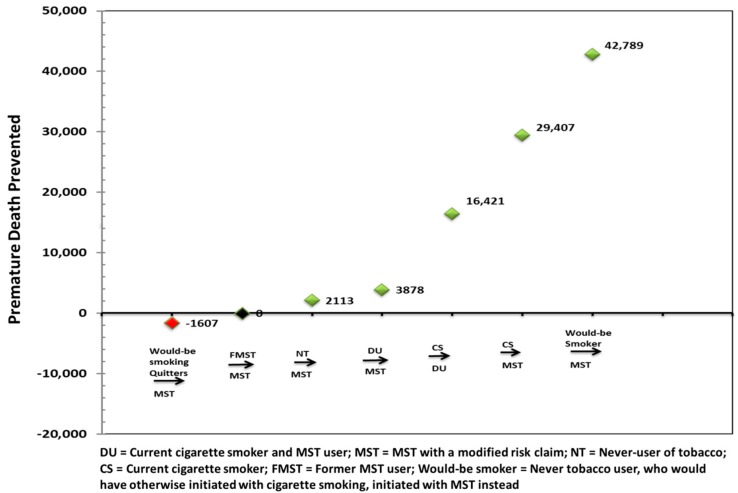
Understanding the impact of the seven key individual transitions.

Figure 3, sorts the individual point estimates in order of ascending benefit, allowing us to understand the contribution of varying transitions. As seen in Figure 3, the majority of the premature deaths prevented (≥95%) are attributed to changes in three product use behaviors: Current cigarette smokers switching to exclusive MST use; current cigarette smokers moving to dual use of cigarettes and MST; and “would-be smokers”, i.e., never tobacco users, who would have otherwise initiated on cigarette smoking, but initiate on the lower risk MST product instead. Although dual use and cigarette smoking states are assigned similar mortality risks in the model, an increase in the premature deaths prevented from current cigarette smokers transitioning to dual use may appear counterintuitive. As discussed above, for the scenario in which cigarette smokers become dual users, we applied the modified case transition rate to that transition and kept all other transitions the same as the base case. Since this modified case transition rate is higher than its corresponding base case transition rate for this transition, it results in a higher number of smokers transitioning to the dual use state versus staying as exclusive smokers. This presents a larger pool of dual users, some of whom move on to become exclusive smokeless tobacco users, which translates to an increase in the number of premature deaths prevented. Furthermore, this transition is supported by observations reported in the literature. Wetter et al. [22] showed that a person who dual uses (i.e., Cigarettes and MST) has an increased probability of transitioning to the exclusive MST use state (i.e., lower relative risk state) (17.4% over a 4-year follow-up), as compared to the probability of an exclusive smoker directly transitioning to exclusive MST use (1.4% over 4-year follow-up).

### 3.3. Sensitivity Analysis

**Figure 4:**
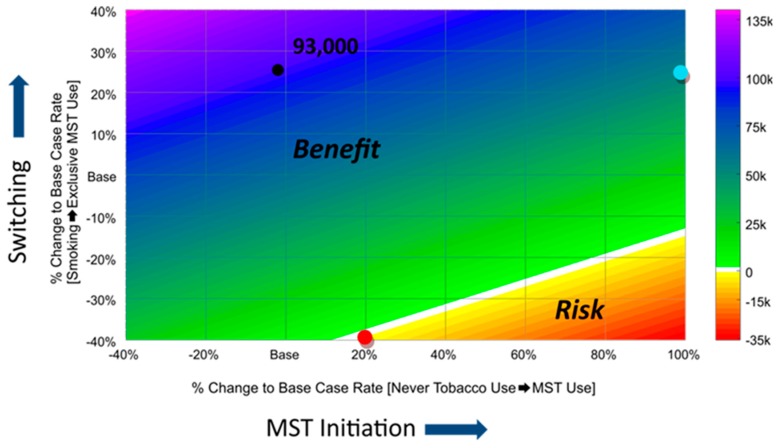
Impact of concurrently varying rates of “Never-Tobacco Users Initiating Exclusive MST Use” and “Cigarette Smokers Switching to Exclusive MST Use” on number of premature deaths prevented. The black dot represents the net benefit of ~93,000 premature deaths prevented over a 60-year period following introduction of the modified risk claim on MST that we observed in our most-likely case scenario, where % changes to the Base Case MST initiation and switching rates were 5% and 21%, respectively. Base indicates the Base Case transition rate, which represents a 0% change in the Modified Case. The values on the bar indicate premature deaths prevented in the different Modified Cases compared to the Base Case. The white line represents the net neutral line (i.e., no difference in premature deaths between Base and Modified Case scenarios), while the green and orange/red areas represent the benefit and risk areas, respectively.

We note that actual use behavior under real-world conditions might yield different transition rates relative to those derived from the CCI Study, measured under a pre-market setting based on exposure to the modified risk claim. Therefore, we conducted a sensitivity analysis to determine the impact of varying key transition rates. For example, one scenario included varying the transition rate of current smokers switching to the MST product over a range of 0.84–1.96% (representing an increase or decrease by 40% of the Base Case transition rate of 1.4%). Another scenario included varying the initiation rate of never tobacco users adopting the MST product from 0.96–3.2% (representing an increase of 100% or decrease of 40% of the Base Case transition rate of 1.6%). We assessed the sensitivity of the multi-cohort model by concurrently varying input rates for the two transition rates while holding all other input transition rates as they are in the Modified Case. Figure 4 illustrates a comprehensive visual summary of the sensitivity analysis and provides insights into how combinations of these parameters impact population level outcomes, allowing for a risk–benefit analysis.

Figure 4 illustrates that the number of premature deaths prevented varies with changes in specific transition rates. The net benefit could only be offset in a highly unlikely scenario, where if the rate of smokers switching to the MST product decreased by 40% (reducing from 1.4–0.84%) and initiation rates simultaneously increased by 20% (increasing from 1.6–1.92%), keeping all other transition rates the same as those in the Modified Case scenario (i.e, represented by the red dot). We do not expect dramatic increases in initiation of ST use from authorization of the modified risk claim. Our analysis of the CCI Study presented in Table 1 indicates that the presence of the claim did not significantly impact the intentions of never tobacco users to initiate MST use. Based on data from the National Survey on Drug Use and Health [43], prevalence of past 30-day ST use among 12–17 year olds has declined from 2% in 2014 to 1.3% in 2017. In addition, our sensitivity analysis shows that even if the initiation rate increases to 100% (i.e., increases from 1.6–3.2%), and holding all other transition rates the same as in the Modified Case, a net benefit of 52,214 premature deaths prevented would still be observed as depicted by the light blue dot. Furthermore, rates of smokers switching to MST products could, at best, remain unchanged or possibly increase when smokers consider the benefit of switching from cigarettes to the lower risk MST product. Therefore, our sensitivity analysis indicates that the net benefit to the population is the likely outcome even if there were “reasonable” deviations in the transition rates.

## 4. Conclusions

We have developed and validated a population model using well-established best modeling practices and tested it using uncertainty and sensitivity analyses. The model was validated by comparing model predictions for the Base Case scenario against published U.S. Life Table (for single-cohort) and U.S. Census Bureau (for multiple-cohort) estimates. The predicted results at all time points were within 6% of the published estimates. The time-staggered multiple-cohort model predicts that authorization of a modified risk claim on an existing MST product will result in a net benefit on the population as a whole, by preventing ~ 93,000 premature deaths within the U.S. male population in 60 years.

The predictions from our model estimations should be considered in the context of some limitations. The temporal resolution of the compartmental model is five years. Hence, it cannot adequately account for participant transitions occurring within a five-year period. We believe that this limitation should not significantly impact the mortality outcomes, as most tobacco-related diseases manifest from chronic use of the product over several decades. Predicting the impact of the reduced risk claim on actual use behavior is difficult in a pre-market setting. Thus, as previously discussed, the relative percent difference in the positive affect measure between the Test and Control Groups within our CCI Study was used to provide a proxy estimate of the expected change in product use behaviors, if the proposed claim was approved. Additionally, transition rates for two of the subpopulations, would-be smokers and would be quitters switching to the MST product, were difficult and impractical to assess in a pre-market setting. In response to this dilemma, we conducted a sensitivity analysis by varying these transition probabilities over a wide range, which demonstrated no indication of unintended consequences completely offsetting the net benefit.

The systematic review by Tam et al. from which we estimated transition rates between cigarette and smokeless tobacco use was based on studies conducted in the 2000 timeframe. We acknowledge that the lack of contemporary data detailing transition probabilities may be a limitation for modeling. A more recent analysis by Chang et al. [44] compared the patterns of transitions between cigarettes and smokeless tobacco use in longitudinal cohorts from 2002–2003 and 2010–2011, from the Tobacco Use Supplement of the Current Population Survey (TUS-CPS). They reported that cigarette smoking cessation had increased in the 2010–2011 cohort, in general the transitions rates between the different smoking and smokeless tobacco use states were fairly consistent between the two cohorts. This further supports our use of transition rates derived from Tam et al. [20]. As with any modeling exercise, we acknowledge that there is the opportunity to update our transition probabilities as more recent data becomes available. For example as more waves of PATH data are released we should be able to obtain a more current set of transition probabilities between cigarettes and smokeless tobacco use in the presence of other tobacco products, particularly e-cigarettes.

The lack of consideration of other tobacco products, particularly with the increased use of e-vapor products over the past few years and their impact on introducing a modified risk claim on an existing smokeless tobacco product may be considered as a limitation. However, the transitions to e-vapor products are dynamic and in flux at the current time. As more waves of PATH data become available, long term established use of e-vapor could yield stable transition rates to different tobacco products. Additionally, the prevalence of current cigar smoking is relatively low, around 2.3% among US adults [45]. Our rationale for only including cigarettes and smokeless tobacco in the base case are as follows. First, cigarette smoking is the most harmful of all tobacco products causing serious and fatal diseases [2]. We included smokeless tobacco as a second tobacco product in the base case since the intent of the model was to assess the impact of switching from cigarettes to a smokeless tobacco product with a proposed modified risk claim. Second, due to the long term use of both cigarettes and smokeless tobacco in the U.S., reliable transition rates between different populations included in the model are well established and available in the peer-reviewed literature. Furthermore, our inclusion of two-products in the base case is a relative improvement compared to the single product models frequently reported in literature, which include cigarettes as the only tobacco product in the their base case, [8,9,11,12,13,14,15,16]. Finally, this approach follows the principle of parsimony described in the Good Modeling Research Practices Overview [17]. A publication by the Board on Population Health and Public Health Practice at the Institute of Medicine further states that “Parsimony allows effective tracing from inputs via specific mechanisms to outputs of interest (giving clear answers to “why” and “how” key results obtain)” [46]. In the context of the current data we believe that our model provides relatively more robust estimates, since we use well-established and stable transition rates of cigarettes and tobacco products.

In conclusion, our computational model comparing well-defined Modified and Base Case scenarios is a validated tool for predicting the net population benefit due to authorization of a modified risk claim on an existing MST product.

## Figures and Tables

**Table 1:** Estimated change in behavioral intentions based on exposure to advertisement with a modified risk claim.

Tobacco Use Transition	Likelihood of Behavior				Relative Percentage Change ^c^
	Control ^a^		Test ^b^		
	Pre ad	Post ad	Pre ad	Post ad	
Never tobacco user ^d^ → candidate MST (Initiation)	3.06%	2.42%	4.85%	3.65%	−5%
Current smoker ^e^ → exclusive use of the candidate MST (Switching)	17.73%	15.87%	13.99%	15.13%	21%
Current smoker → dual use (candidate MST & cigarettes)	24.04%	19.90%	17.86%	18.33%	24%
Dual user (MST & cigarettes) → exclusive use of the candidate MST	34.14%	32.38%	35.48%	35.57%	6%

Note: There were insufficient participants in the Former MST group to obtain a reliable estimate of relapse rates in the control and test group; ^a^ Exposure to ad material without a label claim; ^b^ Exposure to ad material with a label claim; ^c^ Relative percent change = $\frac{\frac{Test\;Post\;Ad}{Test\;Pre\;Ad}-\frac{Control\;Post\;Ad}{Control\;Pre\;Ad}}{\frac{Control\;Post\;Ad}{Control\;Pre\;Ad}};$ ^d^ Never tobacco users are ever-past triers of tobacco that did not reach the smokers lifetime criteria (smoked 100 or more cigarettes) or never-trier of tobacco; ^e^ Current cigarette smokers consist of those both planning and not planning to quit.

**Table 2:** Comparison between Base Case and Modified Case scenarios.

Age (y)	Mean Number of Survivors (Base Case)	Mean Number of Survivors (Modified Case)	Mean Difference in Number of Survivors between Modified Case & Base Case (Premature Deaths Prevented)	95% Credible Interval
43	954,680	954,754	74	(64, 85)
48	931,920	932,117	197	(174, 221)
53	902,538	902,907	369	(324, 417)
58	865,346	865,929	583	(507, 665)
63	817,980	818,792	812	(700, 936)
68	756,831	757,842	1010	(866, 1169)
73	676,903	678,023	1120	(958, 1301)

Note: In the table, results are reported only for ages 43–73. In the model, survivability of the initial cohort of 1,000,000 males is followed in five-year intervals, starting from age 13.

**Table 3:** Estimated additional years of expected life between the Base Case and Modified Case scenarios.

Age (y)	Modified Case Scenario Life Table		Base Case Life Table		Difference (Modified Case–Base Case)	
	*l_x(m)_* ^1^	*T_x(m)_* ^2^	*l_x(b)_* ^3^	*T_x(b)_* ^4^	*l_x(m−b)_* ^5^	*T_x(m−b)_* ^6^
13	1,000,000	59,914,223	1,000,000	59,881,367	0	32,856
18	997,317	58,912,882	997,317	58,880,026	0	32,856
23	993,963	53,934,688	993,963	53,901,832	0	32,856
28	989,041	48,977,188	989,036	48,944,346	5	32,843
33	981,605	44,050,596	981,594	44,017,793	11	32,803
38	970,653	39,170,002	970,627	39,137,291	26	32,711
43	954,754	34,356,594	954,680	34,324,134	74	32,461
48	932,117	29,639,644	931,920	29,607,864	197	31,780
53	902,907	25,052,474	902,538	25,022,113	369	30,361
58	865,929	20,631,030	865,346	20,603,055	583	27,975
63	818,792	16,420,329	817,980	16,395,852	812	24,477
68	757,842	12,480,710	756,831	12,460,804	1010	19,906
73 +	678,023	8,894,749	676,903	8,880,185	1120	14,564

Note: The life table underlying this extends to a final open-ended age group of 103 + years. The values shown above in this table for 73 + years are calculated from the underlying life table; ^1^ *l_x(m)_* = expected number of survivors at the start of each age group in the Modified Case; ^2^ *T_x(m)_* = expected cumulative number of years of life remaining for the survivors at the start of each age group, x in the Modified Case; ^3^ *lx_(b)_* = expected number of survivors at the start of each age group, x in the Base Case; ^4^ *T_x(b)_* = expected cumulative number of years of life remaining for the survivors at the start of each age group, x in the Base Case; ^5^ *l_x(m−b)_* = expected number of additional survivors at each age group, x in the Modified Case versus the Base Case; ^6^ *T_x(m−b)_* = expected cumulative number of additional years of life lived by the additional survivors at the start of each age group, x in the Modified Case versus the Base Case.

**Table 4:** Difference in the number of survivors between the Modified and Base Case scenarios at the end of a 60-year follow-up post authorization to introduce a modified risk claim on the candidate smokeless tobacco product.

Age Group (years)	Master Case	Base Case	Difference
0–4	11,659,500	11,659,500	0
5–9	11,503,227	11,503,227	0
10–14	11,343,808	11,343,808	0
15–19	11,384,863	11,384,863	0
20–24	11,210,354	11,210,354	0
25–29	10,975,495	10,975,342	153
30–34	10,691,665	10,691,192	473
35–39	10,398,367	10,397,394	973
40–44	10,101,332	10,099,412	1920
45–49	9,787,295	9,783,564	3731
50–54	9,355,425	9,348,637	6788
55–59	8,757,301	8,747,530	9771
60–64	8,050,922	8,038,615	12,307
65–69	7,691,177	7,676,364	14,813
70–74	6,889,508	6,873,894	15,614
75–79	5,774,009	5,759,539	14,470
80–84	4,761,915	4,749,605	12,310
Total additional number of survivors in the Modified vs. Base Case			93,323

## Supplementary Materials

The following are available online at https://www.mdpi.com/1660-4601/16/7/1264/s1, Supplementary File 1: Model Development, Supplementary File 2: Input Data, Supplementary File 3: Model Validation.
