# Supplementary material for: A Computational Model for Assessing the Population Health Impact of Introducing a Modified Risk Claim on an Existing Smokeless Tobacco Product
Source: Int J Environ Res Public Health. 2019 Apr 9;16(7):1264. doi: 10.3390/ijerph16071264 (PMC6479389; doi:10.3390/ijerph16071264)
Supplement: Supplementary file 1 [file ijerph-16-01264-s001.zip › Supplementary File 1-updated_03142019.pdf]

A Computational Model for Assessing the Population Health Impact of Introducing a Modified Risk Claim on an Existing Smokeless Tobacco Product

Raheema S. Muhammad-Kah, Yezdi B. Pithawalla, Edward L. Boone, Lai Wei, Michael A. Jones, Ryan A. Black, Thomas M. Bryan, Mohamadi A. Sarkar

Supplementary File 1. MODEL DEVELOPMENT

1.1. Transition model

The Markov chains approach we implemented in the single cohort model starts with a hypothetical cohort of one million males, with survival estimated in five-year intervals, starting from age 13 years. The membership of each state was determined. The following states were considered:

- Never-User of Tobacco (NT)
- Current Cigarette Smoker (CS)
- Former Cigarette Smoker (FCS)
- Current MST User (MST)
- Former MST User (FMST)
- Dual User (DU) (Current Cigarette Smoker and Current MST User)
- Former Dual User (FDU) (Both former Cigarette Smoker and former MST User, but currently uses neither product)

To assess these states, we had to consider the paths that a participant (cohort member) could progress along between these states. Figure S1.1 shows the states, transitions, and equations used to calculate the relevant transition probabilities between the different states for the compartmental model used. The pathways that an individual could progress through result in 29 states. There are 30 input parameters used to calculate the transition probabilities. This results in a vector of 29 states and a 29 × 29 transition matrix.

Figure S1.1: Compartmental Model with States and possible pathways

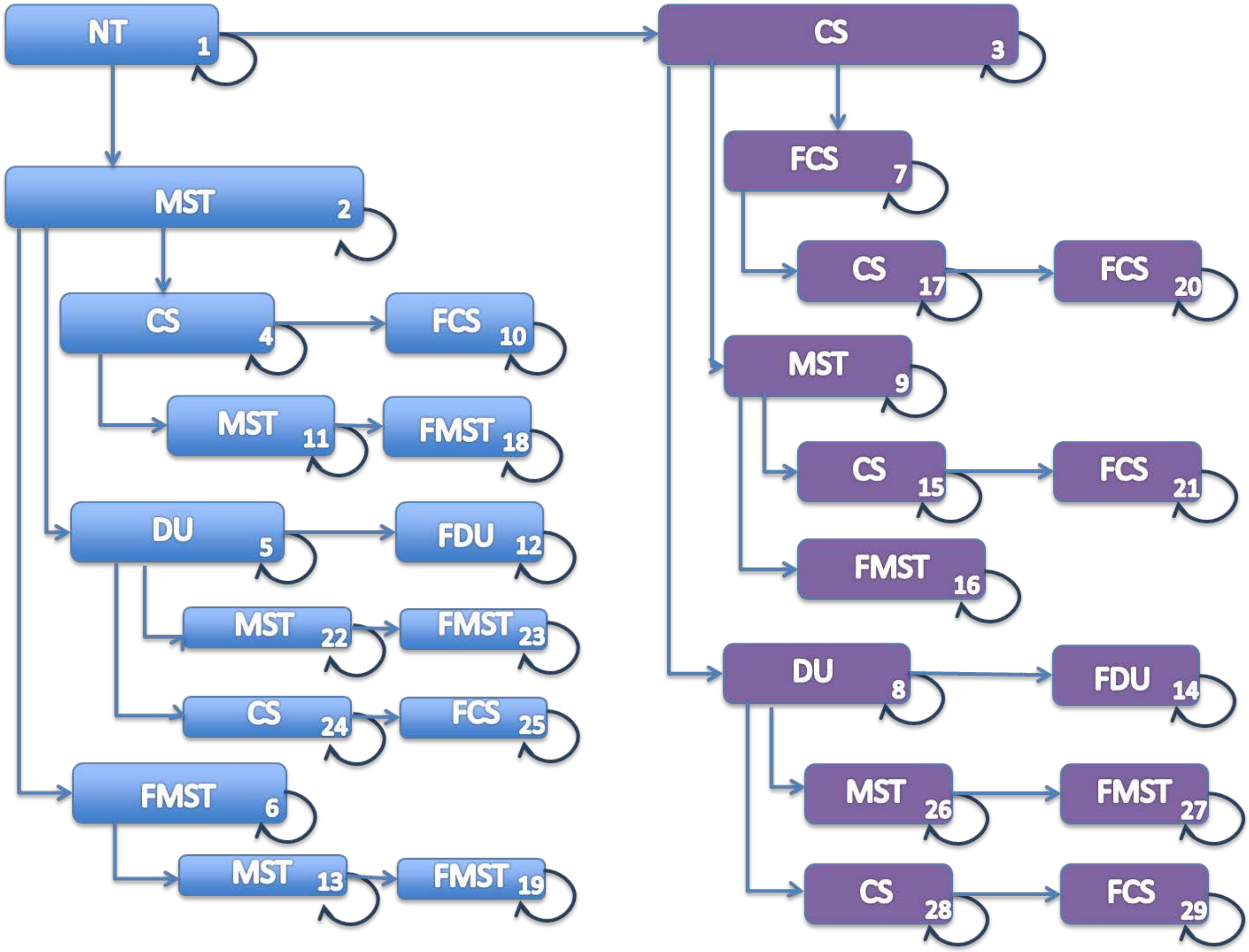

Note: The numbers in the right bottom of each box represents the corresponding State number (e.g., State 1 is an NT state, while State 17 is a CS state).
CS = current cigarette smoker; DU = dual user; FCS = former cigarette smoker; FDU = former dual user; FMST = former MST user; MST = current MST users; NT = never-user of tobacco.

1.2. Time-staggered Multiple Cohort Approach

To employ a multiple-cohort modeling approach, it is imperative that the combination of the single cohorts resembles the population of interest. To achieve this, each age-group cohort must be created at birth and be moved forward through time using the single-cohort model (Figure S1.2). Once enough single-cohort groups are developed, the combination of these single-cohort groups should resemble the population of interest, provided mortality and birth rates are adequately specified. Each age group needs specific tobacco initiation and cessation rates and other transition probabilities through time in order to account for variation in these rates at different ages. In addition, a robust mortality model needs to be employed so that each cohort's estimated mortality rates adequately reflect the true mortality rates.

Figure S1.2. Time-Staggered Multiple Cohort Approach

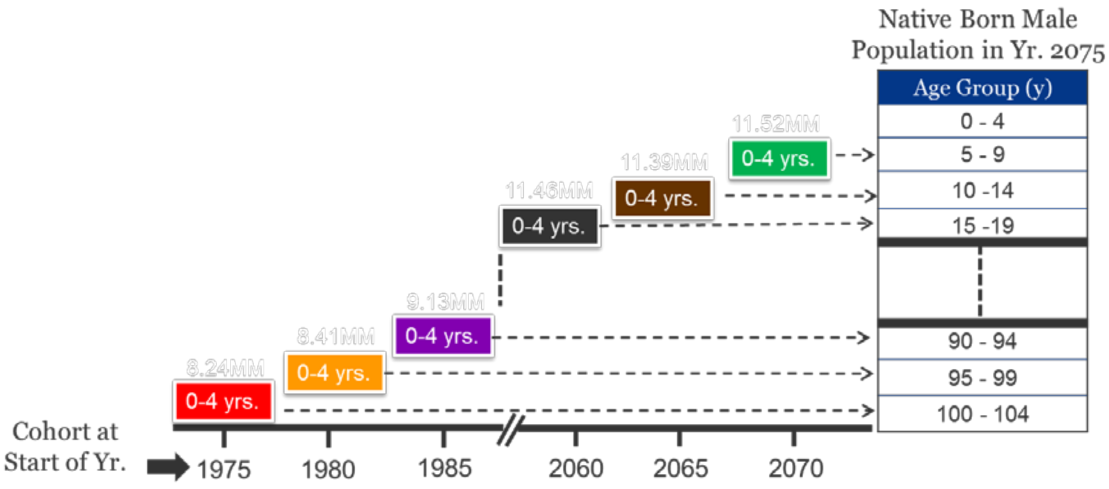

1.3. Mortality model

To model mortality associated across age, we developed Poisson regression models. We estimated the parameters in the model using a Bayesian approach via the Markov Chain Monte Carlo (MCMC) procedure from the MCMCpack package in the R statistical programming environment. The goal of the Bayesian analysis was to determine the posterior distribution for the parameters. For notation, let $p(\beta)$ be the prior distribution for the parameter β, and let $L(D|\beta)$ be the likelihood of data D, given the parameter β. To obtain the posterior distribution $p(\beta|D)$, we used Bayes theorem denoted below:

$$p(\beta|D) = \frac{p(\beta)L(D|\beta)}{\int p(\beta)L(D|\beta)d\beta}$$

Often, the posterior distribution is not analytically tractable; hence, the posterior distribution must be explored using computer sampling. By taking a large number of samples from the posterior distribution, one can use sample quantities to make inferences. The MCMCpack in R utilizes the Metropolis-Hastings Algorithm to obtain samples from the posterior distribution.

To estimate mortality related parameters from the authorization of the proposed claim, the model includes the following use states: NT; CS; FCS; Current MST Users (MST); Former MST Users (FMST); Dual Users (DU) (i.e., both current cigarette and MST users); and Former Dual Users (FDU). We employ the concept of Excess Relative Risk (ERR) ratio to adjust the risk associated with smoking and to approximate the risks associated with both use of exclusive MST and dual use (See Supplementary File 2). Using ERR ratios and mortality risk related to NT, CS and FCS use, we are able to derive the accumulated risks associated with spending time in each state based on a cohort members specific pathway.

Table Supplementary File 1.1: Derivation of Mortality Rates

State (s)/ Pathway	Risk Formula
Never tobacco user	$r_{NT}(age)$
MST user	$r_{MST}(Age) = r_{NT}(Age)* [\mathrm{ERR}_{(MST\|CS)} * RR_{CS}(Age) + (1 - \mathrm{ERR}_{(MST\|CS)})]$
Current Smoker	$r_{CS}(Age) = r_{NT}(Age) * RR_{CS}(Age)$
MST user who switch to Cigarette Smoking	$r_{MST\ to\ CS}(Age) = r_{NT}(Age) * RR_{CS}(Age) * RR_{MST}(Age)$
Dual user	$r_{DU}(Age) = RR_{DU}(Age) * r_{CS}(Age)$
Former MST user	$r_{FMST}(Age) = r_{NT}(Age) * \mathrm{ERR}_{(FMST\|FS)} * RR_{FS}(Age) * RR_{CS}(Age) + (1 - \mathrm{ERR}_{(FMST\|FS)}) * r_{NT}(Age)$
Former Smoker	$r_{CSFS}(Age) = r_{NT}(Age) * RR_{CS}(Age) * RR_{FS}(Age)$
Cigarette Smoker who switch to MST use	$r_{CS\ to\ MST}(Age) = r_{NT}(Age) * RR_{CS}(Age) * [\mathrm{ERR}_{(MST\|CS)} * RR_{CS}(Age) + (1 - \mathrm{ERR}_{(MST\|CS)})] * [\mathrm{ERR}_{(MST\|CS)} * RR_{FS}(Age) + (1 - \mathrm{ERR}_{(MST\|CS)})]$
MST user who switch to Cigarette Smoking then Quit	$r_{MST\ to\ CS\ to\ FS}(Age) = r_{NT}(Age) * RR_{CSFS}(Age) * RR_{MST}(Age)$
Former Dual user	$r_{FDU}(Age) = RR_{FDU}(Age) * r_{CSFS}(Age)$
Cigarette Smoker who switch to MST use then Quit	$r_{CS\ to\ MST\ to\ FMST}(Age) = r_{NT}(Age) * RR_{CS}(Age) * RR_{FMST}(Age) * [\mathrm{ERR}_{(MST\|CS)} * RR_{CSFS}(Age) + (1 - \mathrm{ERR}_{(MST\|CS)})]$

Note that :

r = mortality rate, ERR= excess relative risk ratio, RR= Relative risk vs background risk

$r_{NT}(age) = \beta_0 + \beta_1 AGE_i + \beta_2 AGE_i^2$ is the mortality rate for Never Users of Tobacco
$r_{CS}(age) = \beta_0 + \beta_1 AGE_i + \beta_2 AGE_i^2 + \beta_3 YSM_i + \beta_4 YSM_i \times AGE_i$ is the mortality rate for Current Cigarette Smoker
$r_{CSFS}(age) = \beta_0 + \beta_1 AGE_i + \beta_2 AGE_i^2 + \beta_3 YSM_i + \beta_4 YQSM_i + \beta_5 YSM_i \times AGE_i + \beta_6 YSQM_i \times AGE_i$ is the mortality rate for Former Cigarette Smokers
$RR_{CS}(age) = \frac{r_{CS}(age)}{r_{NS}(age)}$ is the relative risk of cigarette smoking
$RR_{MST}(Age)$ = $\text{ERR}_{(\text{MST}|\text{CS})} * RR_{CS}(Age) + (1 - \text{ERR}_{(\text{MST}|\text{CS})})$ is the relative risk of MST use
$RR_{DU}(Age)$ = [$ERR_{DU}(Age)$* ($RR_{CS}(Age)$ -1) +1] / $RR_{CS}(Age)$ = relative risk of dual use
$RR_{FS}(Age) = \frac{r_{CSFS}(Age)}{r_{CS}(Age)}$ is the relative risk of former cigarette smoking
$RR_{FMST}(Age)$ = $\text{ERR}_{(\text{FMST}|\text{FS})} * RR_{CS}(Age) * RR_{FS}(Age) + (1 - \text{ERR}_{(\text{FMST}|\text{FS})})$ is the relative risk of Former MST use
$RR_{FDU}(Age)$ = [$ERR_{FDU}(Age)$* ($RR_{FS}(Age)$ -1) +1] / $RR_{FS}(Age)$ is the relative risk of Former dual use

1.4. Model PseudoCode

```
###### Pseudocode to run the ALCS Cohort model with a Single Cohort#######

# Specify the size of the cohort
n1 <- 1000000          # Number of individuals in a single cohort
# Define the number of MCMC samples to pull and how many samples to use as burn-in samples.
nmcmc1 <- 10000
burn <- 2000

##################################################################
# Trans1fn, which creates the transition matrix for the Markov model
# Input:   M1 is a vector of length 30 containing the transition probabilities for a specific age group this can be modified depending on what age group you would like to account for.
# Output:  Trans1a is a 29 by 29 matrix corresponding to the Markov chain
###################################################################

Trans1fn <- function (M1)
{
        Initialize transition matrix Trans1a (a 29X29 matrix)
        Read input transition matrix M1 to fill in transition matrix Trans1a
        Return (Trans1a)
}
# Create a base case for the transition probabilities
# M1 through M12 are found in a file source.
# M1b <- rbind (M1, M2, M3, M4, M5, M6, M7, M8, M9, M10, M11, M12)
# where M1 = age group 13-17, M2 = age group 18-24,…………. etc
# Create a modified case for transition probabilities
# These probabilities reflect the impact of the changes observed in the modified case
# M1m <- rbind(M1, M2, M3, M4, M5, M6, M7, M8, M9, M10, M11, M12)
############################################################
# Assign Excess Relative Risk Ratio (ERR) Values
############################################################
ERRmrtp1         <- 0.09     # ERR for the MST versus CS
ERRmrtpfmrtp1 <- 0.04     # ERR for the Former MST
ERRduVcs1        <- 1          # ERR for DU versus CS
ERRfduVfcs1     <- 1          # ERR for FDU versus FCS

############################################################
# Build mortality models
############################################################
# Read in the mortality data as MT1 including Age, Smoking Status, Years of Smoking (YSM), Years since quit smoking (YQSM), Person Years(PY), Number of Death(NoD), Mortality per Person Year (MRP)

Read MT1 from CSV file

# Create the additional variables needed in the models (transformations of existing variables)
MT1 <- Combine MT1 with Age^2 (Age2), Number of Deaths (NoD)*10, Person Years (PY)*10
Subset the data into three individual datasets (never smoker (MT1.ns), cigarette smoker (MT1.cs), former smoker (MT1.csfs)) for each model.
```

```
# Obtain starting values for the never tobacco, cigarette smoker, former smoker mortality model using maximum likelihood through GLM
MT1.glm.ns <- GLM model MRP*100 ~ AGE + AGE2 with family=poisson(link=log)
MT1.glm.cs <- GLM model MRP*100 ~ AGE + AGE2 + YSM + YSM: AGE, with family=poisson(link=log)
MT1.glm.csfs <- GLM model MRP*100 ~ AGE + AGE2 + YSM + YQSM + AGE: YSM + AGE: YQSM with family=poisson(link=log)

# Obtain the samples (n=10000) from the MCMC sampler for the never tobacco, cigarette smoker, former smoker  mortality model with 2000 burn-in samples
one1.ns <- MCMC samples based on model MRP*100 ~ AGE + AGE2 with poisson(link=log).
one1.cs <- MCMC samples based on model MRP*100 ~ AGE + AGE2 + YSM + AGE:YSM, with family=poisson(link=log).
one1.csfs <- MCMC samples based on model MRP*100 ~ AGE + AGE2 + YSM + YQSM + AGE:YSM + AGE:YQSM, family=poisson(link=log).

################################################################################
# Set up the dataset including YSM, YQSM for the population for the predictions
################################################################################
# Never Smoker
new3ns0 <- data frame with three columns: intercept (with value 1), age, age^2
# Cigarette smoker
new3cs0 <- data frame with five columns: intercept (with value 1), age, age^2, YSM, Age*YSM)
#Former Smoker
new3csfcs0 <- data.frame with seven columns: intercept (with value 1), age, age^2, YSM, YQSM,  Age*YSM, AGE*YQSM)

################################################################################
# Loop through all the MCMC samples
################################################################################
for (k1 in 1:nmcmc1){
        ##############################
        # Calculate Survival Rates
        ##############################
        #Survival rate of never smoker
        r.ns0 <- Generate the risk amount from Poisson distribution based on mean risk rate with lambda= exp(new3ns0*one1.ns)
        s.ns0 <- Convert to survival rate for never tobacco (1 - r.ns0)
         #Survival rate of cigarette smoker
        r.cs0 <- Generate the risk amount from Poisson distribution based on mean risk rate with lambda= exp(new3cs0*one1.cs)
        RR.cs0 <- Generate the relative risk rate for cigarette smokers (r.cs0/r.ns0)
        s.cs0 <- Convert to survival rate for cigarette smokers (1- r.cs0)
        #Survival rate of former smoker
        r.csfs0 <- Generate the risk amount from Poisson distribution based on mean risk rate with lambda=exp(new3csfcs0*one1.csfs)
        RR.csfs0 <-  Bound the relative risk rate for former cigarette smokers by restricting r.csfs0/r.ns0 < 1.05
        s.csfs0 <- Convert to survival rate for cigarette smokers (1- r.csfs0)
        #Survival rate of MRTP user
        RR.mrtp0 <- Generate relative risk for MRTP use relative to cigarette smokers based on ERR
        r.mrtp0 <- Generate the risk of MRTP use
        s.mrtp0 <- Convert to survival probability for MRTP users (1- r.mrtp0)
        ##### Following the same steps and formulas provided in Supplementary File 1 (Section 1.3)  we generate survival rates for former MRTP user (s.mrtpfmrtp0), dual user (s.duVcs0), former dual user (s.fduVfcs0), survival rates for individuals who transitioned from cigarette smoking to MRTP use (s.csmrtp0), survival rates for individuals who transitioned from MRTP use to cigarette smoking (s.mrtpcs0), survival rates for individuals who transitioned from
```

cigarette smoking to MRTP use and then quit (csmrtpfmrtp0) and survival rates for individuals who transitioned from MRTP use to cigarette smoking and then quit (s.mrtpcsfs0)

```
##############################
# Loop through age groups
##############################
For (i in five year age groups){
index1 <- # Assign the ages in the age groups indexes
Calculate 5 year survival probabilities age intervals (s.ns1, s.cs1, s.csfs1, s.mrtp1, s.mrtpfmrtp1, s.csmrtp1, s.csmrtpfmrtp1, s.mrtpcsfs1, s.du1, s.fdu1) based on survival rates calculated above
 Assign survival probabilities to 29 states of the model
# Pass the current cohort through the survival step
state1b <- state1b*SurvState1
state1m <- state1m*SurvState1
#Pass the current cohort through the transition step
state1b <- state1b%*%Trans1fn(M1ba)
state1m <- state1m%*%Trans1fn(M1ma)

}
Combine the results through multiple age groups to output
}
```
