# Supplementary material for: A Computational Model for Assessing the Population Health Impact of Introducing a Modified Risk Claim on an Existing Smokeless Tobacco Product
Source: Int J Environ Res Public Health. 2019 Apr 9;16(7):1264. doi: 10.3390/ijerph16071264 (PMC6479389; doi:10.3390/ijerph16071264)
Supplement: Supplementary file 1 [file ijerph-16-01264-s001.zip › Supplementary File 2-20190131.pdf]

# A Computational Model for Assessing the Population Health Impact of Introducing a Modified Risk Claim on an Existing Smokeless Tobacco Product

Raheema S. Muhammad-Kah, Yezdi B. Pithawalla, Edward L. Boone, Lai Wei, Michael A. Jones, Ryan A. Black, Thomas M. Bryan, Mohamadi A. Sarkar

## Supplementary File 2. INPUT DATA

## 2.1. Transition data

### 2.1.1. Base Case Transitions Estimated from Published Literature

Tam et al. [1] conducted a systematic review of published literature on transitions between smokeless tobacco (ST) and cigarette use in the United States. The reviewers discussed six studies of U.S. populations [2-4], wherein longitudinal data were presented on some or all of the transitions that users can undergo between never-tobacco use, ST use, cigarette use, and dual use states.

We used transition rates from three of these studies [2-4] because the underlying populations assessed in these studies were the most generalizable to U.S. population, male specific transition rates were measured and the transition periods were most similar to those used in our modeling analyses.

- Male adolescent transitions
  - Tomar [2] reported transition probabilities from the Teenage Attitudes and Practices Survey-I and Teenage Attitudes and Practices Survey-II (TAPS-I and II), which were nationally representative cohort studies conducted in 1988/1989 and 1993 and included 3,996 males with ages ranging from 11 to 19 years. This study had a 4-year follow-up between 1989 and 1993.
- Adult male transitions
  - Wetter et al. [3] studied secondary trial data from the Working Well Trial on cancer prevention, which included 1,224 adult male tobacco users residing in the southeastern United States, with baseline in 1990 and follow-up 4 years later. The average age of the participants in this study was 37.5 years.
  - Zhu et al. [4] reported the results from the 2002 Tobacco Use Supplement to the Current Population Survey, a nationally representative cross-sectional survey including both males and females over the age of 18 years. Follow-up interviews for 15,056 households were conducted in 2003.

Based on our analysis of the information presented within Tam et al. [1] and the associated references, we summarize the male adolescent Base Case transition probabilities in Table S2.1 based on the 4-year follow up TAPS-I and II study by Tomar [2]. We make the assumption that the 4 year follow up data can be used as model inputs for 5-year estimates.

We further summarized the male adult Base Case transition probabilities based on the 1-year follow-up study by Zhu et al. [4] and the 4-year follow-up study by Wetter et al. [3] in Table

S2.2. For 1 year follow-up data, we adjusted the yearly rates to obtain rates in appropriate for our 5 year increments. This adjustment involves multiplying the transition probability by 2.5, the average person-time at risk of smoking initiation in each 5 year age category.

**Table S2.1 Percentage of Male Adolescents Transitioning between Tobacco Product Use Categories: 4-Year Follow-up [2]**

| Baseline Status | Follow-up Status | | | |
|---|---|---|---|---|
| | Neither | Exclusive Smokeless Tobacco User | Exclusive Smoker | Dual User |
| Neither | 82.2% | 3.1% | 13.5% | 1.1% |
| Exclusive smokeless tobacco user | 15.2% | 44.8% | 25.5% | 14.3% |
| Exclusive smoker | 16.9% | 0.8% | 78.7% | 3.6% |
| Dual user | 14.1% | 34.2% | 31.2% | 20.4% |

Note: Due to the effect of rounding, not all percentages sum exactly to 100.

**Table S2.2: Percentage of Male Adults Transitioning Between Tobacco Product Use Categories (1-Year Follow-Up from Zhu et al. [4] and 4-Year Follow-Up from Wetter et al. [3])**

| Baseline Status | Follow-up Status | | | |
|---|---|---|---|---|
| | Neither | Exclusive Smokeless Tobacco User | Exclusive Smoker | Dual User |
| Neither | 96.7%<br>1-year follow-up | 0.7%<br>1-year follow-up | 2.5%<br>1-year follow-up | 0.1%<br>1 year follow-up |
| Exclusive smokeless tobacco user | 20.1%<br>4-year follow-up | 76.6%<br>4-year follow-up | 0.9%<br>4-year follow-up | 2.5%<br>4-year follow-up |
| Exclusive smoker | 15.7%<br>4-year follow-up | 1.4%<br>4-year follow-up | 79.7%<br>4-year follow-up | 3.2%<br>4-year follow-up |
| Dual user | 11.3%<br>4-year follow-up | 17.4%<br>4-year follow-up | 27.0%<br>4-year follow-up | 44.3%<br>4-year follow-up |

Note: Due to the effect of rounding, not all percentages sum exactly to 100.

### 2.1.2. Modified Cases Transitions Estimated from ALCS Claim Comprehension & Intentions Study

We estimate relative percent change in transition rates by evaluating the percent difference between the relevant responses of the test condition (exposed to advertisement with the proposed modified risk claim) and control condition (exposed to advertisement without the proposed claim) from pre to post-ad exposure from the ALCS Claim Comprehension &

Intentions (CCI) Study and then apply the relative percent change factor to the transition rates used in the Base Case, which are obtained from nationally representative studies reported in Tam et al. The methodology applied to estimate these percent differences for all the relevant transitions is discussed below.

The CCI Study is a cross-sectional, self-administered, computerized survey study. The study population was composed of a non-probability sample of qualified adult (legal age to purchase tobacco products or older), self-reported tobacco product users and non-users. The study assesses intentions to try, using, dual use, and switching to the test product among adult self-reported tobacco product users and non-users. The outcomes are extracted for modeling purposes and are not expected to be comparable to the Base Case transition probabilities.

As an example, consider the transition rate for current exclusive smokers transitioning to current exclusive MST use, which is 1.4 percent for the male adult group (as shown in Table S2.2). In order to estimate the percent by which this Base Case transition rates would be changed to calculate the estimated transition rates for the Modified Case, we analyzed the percent difference between the relevant responses of the test condition (exposed to advertisement with the modified risk claim) and control condition (exposed to advertisement with without the modified risk claim) in the ALCS CCIS. To illustrate, consider the example where at pre-ad within the control condition 17.73% of current exclusive smokers intend to switch to the test product, 15.87% intend to switch post-ad, 13.99% of exclusive smokers in the test condition at pre-ad intend to switch to the test product and 15.13% intend to switch at post-ad. The relative percentage change calculation is 21% probability of switching to the test product. That is, the ratio of test condition respondents (post-ad vs pre-ad) minus the ratio of control condition respondents (post-ad vs pre-ad) divided by the ratio of control condition respondents will generate the relative percentage change. Applying this change of 21 percent to the Base Case value of 1.4 percent would make the probability of this transition 1.7 percent.

## 2.2. Mortality data

The data set used to create the mortality models is based on a Kaiser Permanente (KP) Medical Care Program Cohort study. Friedman et al. [5] provided a data set that addresses the relative mortality of current cigarette smokers, former cigarette smokers, and never user of tobacco at various ages. The KP Medical Care Program Cohort study obtained baseline information including age, sex, and smoking status history on more than 60,000 subjects, age 35 years and older and followed the cohort for mortality over a period of time. This is one of the few published data sets available that contain information about how long a person has been a cigarette smoker, whether the person had quit smoking, and, if so, how long the person has been a former cigarette smoker.

The adjustment of the original KP data set includes the following steps:

a. The data were divided into narrower intervals to better align with the age groups used in our modeling application. The mortality rates were calculated by dividing the number of deaths from all causes by person years reported in the KP study. The updated table is shown in Table S2.3.

b. All the subjects in the data set had health insurance, the observation period was short, and the age-specific mortality rates are likely to be lower than that within the U.S. population. Therefore, we adjusted the KP mortality data in Table S2.4 by using the concept of excess mortality ratio (i.e. ratio of mortality rates in the U.S. population to the mortality rates reported in the KP data set).

Table S2.5 reflects the final data set used to estimate the parameters in the mortality models, which includes age, current smoking status, years smoked, years since quit smoking, person-years, number of deaths due to all causes, and calculated mortality rate (i.e., number of deaths/person-years) (nmx). As described in the publication, the ultimate goal is to create three separate mortality models for never-users of tobacco, cigarette smokers, and former cigarette smokers, by fitting their corresponding data sets using the natural log link:

$$MR_i \sim Poisson(\lambda_i)$$

where $MR$ is the mortality rate corresponding to each 100,000 people and $\lambda$ is the mean mortality rate and *i* represents members within each specific user group (Never Users, cigarette smokers and former smokers).

**Table S2.3: Number of Deaths Due to All-Causes and Age Assignments to Subintervals Categorize by Age Group for Male Current Cigarette Smokers**

| Age (y) | Status | Years Smoked | Person Years | Number of Deaths (All-Cause) |
|---|---|---|---|---|
| 35 to 42 | Never | - | 14,958 | 19.6 |
| | Current | 1 to 10 | - | - |
| | Current | 11 to 19 | 2,970 | 6.4 |
| | Current | 20 to 29 | 7,281.5 | 19.2 |
| | Current | 30 to 39 | - | - |
| 43 to 49 | Never | - | 14,958 | 29.4 |
| | Current | 1 to 10 | - | - |
| | Current | 11 to 19 | 2,970 | 9.6 |
| | Current | 20 to 29 | 72,81.5 | 28.8 |
| | Current | 30 to 39 | - | - |
| 50 to 56 | Never | - | 12,010 | 38.8 |
| | Current | 1 to 10 | - | - |
| | Current | 11 to 19 | 1,174 | 7 |
| | Current | 20 to 29 | 5,102.5 | 32 |
| | Current | 30 to 39 | 2,551.3 | 19.2 |
| | Current | 40+ | - | - |
| 57 to 64 | Never | - | 12,010 | 58.2 |

| Age (y) | Status | Years Smoked | Person Years | Number of Deaths (All-Cause) |
|---|---|---|---|---|
| | Current | 1 to 10 | - | - |
| | Current | 11 to 19 | - | - |
| | Current | 20 to 29 | - | - |
| | Current | 30 to 39 | 2,551.3 | 28.8 |
| | Current | 40+ | 4,367 | 74 |
| 65 to 74 | Never | - | 11,466 | 161 |
| | Current | 1 to 10 | - | - |
| | Current | 11 to 19 | - | - |
| | Current | 20 to 29 | - | - |
| | Current | 30 to 39 | 963 | 23 |
| | Current | 40+ | 3,285 | 80 |
| 75+ | Never | - | 4,486 | 203 |
| | Current | 1 to 10 | - | - |
| | Current | 11 to 19 | - | - |
| | Current | 20 to 29 | - | - |
| | Current | 30 to 39 | 138 | 12 |
| | Current | 40+ | 740 | 42 |

## Table S2.4: Mortality Data Set Adjusted to Reflect the General Male United States Population

| Age (y) | Current Smoking Status[1] | Years Smoked | Years Quit | Person Years | Number of Deaths (All-Cause) |
|---|---|---|---|---|---|
| 39 | 1 | 0 | 0 | 14,958 | 19.54 |
| 39 | 2 | 5 | 6 | 1,392.75 | 1.91 |
| 39 | 2 | 5 | 16 | 3,105 | 3.59 |
| 39 | 2 | 15 | 6 | 1,392.75 | 2.87 |
| 39 | 3 | 15 | 0 | 2,970 | 6.38 |
| 39 | 3 | 25 | 0 | 7,281.5 | 19.14 |
| 46.5 | 1 | 0 | 0 | 14,958 | 29.31 |
| 46.5 | 2 | 5 | 6 | 1,392.75 | 2.87 |
| 46.5 | 2 | 5 | 16 | 3,105 | 5.38 |
| 46.5 | 2 | 5 | 26 | 1,149 | 1.99 |
| 46.5 | 2 | 15 | 6 | 1,392.75 | 4.31 |

| Age (y) | Current Smoking Status[1] | Years Smoked | Years Quit | Person Years | Number of Deaths (All-Cause) |
|---|---|---|---|---|---|
| 46.5 | 3 | 15 | 0 | 2,970 | 9.57 |
| 46.5 | 3 | 25 | 0 | 7,281.5 | 28.72 |
| 53.5 | 1 | 0 | 0 | 12,010 | 79.95 |
| 53.5 | 2 | 15 | 16 | 1,526.75 | 9.56 |
| 53.5 | 2 | 15 | 26 | 2,335 | 15.66 |
| 53.5 | 2 | 25 | 6 | 906.25 | 8.57 |
| 53.5 | 2 | 25 | 16 | 1,526.75 | 14.34 |
| 53.5 | 2 | 35 | 6 | 906.25 | 12.86 |
| 53.5 | 3 | 15 | 0 | 1,174 | 14.42 |
| 53.5 | 3 | 25 | 0 | 5,102.5 | 65.94 |
| 53.5 | 3 | 35 | 0 | 2,551.25 | 39.57 |
| 61 | 1 | 0 | 0 | 12,010 | 119.93 |
| 61 | 2 | 15 | 16 | 1,526.75 | 14.34 |
| 61 | 2 | 15 | 26 | 2,335 | 23.49 |
| 61 | 2 | 25 | 6 | 906.25 | 12.86 |
| 61 | 2 | 25 | 16 | 1,526.75 | 21.51 |
| 61 | 2 | 35 | 6 | 906.25 | 19.29 |
| 61 | 3 | 35 | 0 | 2,551.25 | 59.35 |
| 61 | 3 | 40 | 0 | 4,367 | 152.49 |
| 70 | 1 | 0 | 0 | 11,466 | 345.96 |
| 70 | 2 | 25 | 16 | 1,274 | 44.7 |
| 70 | 2 | 25 | 26 | 3,507 | 92.4 |
| 70 | 2 | 35 | 6 | 977 | 30.08 |
| 70 | 2 | 35 | 16 | 1,274 | 67.04 |
| 70 | 3 | 35 | 0 | 963 | 49.42 |
| 70 | 3 | 40 | 0 | 3,285 | 171.91 |
| 80 | 1 | 0 | 0 | 4,486 | 263.32 |
| 80 | 2 | 35 | 16 | 335.5 | 20.75 |
| 80 | 2 | 35 | 26 | 1,442 | 86.91 |
| 80 | 2 | 55 | 6 | 253 | 20.75 |
| 80 | 2 | 55 | 16 | 335.5 | 31.13 |
| 80 | 3 | 35 | 0 | 138 | 15.57 |
| 80 | 3 | 40 | 0 | 740 | 54.48 |

[1] 1 = Never-Tobacco User, 2 = Former Smoker, 3 = Current Smoker

**Table S2.5: Mortality Data Set with Estimated Mortality Rates: Males for Year 2000**

| **Age (y)** | Current Smoking Status[1] | Years Smoked | Years Quit | Person Years | Number of Deaths (All-Cause) | **Mortality Rate ($_nm_x$)** | **MRP** |
|---|---|---|---|---|---|---|---|
| 39 | 1 | 0 | 0 | 14958 | 33.3 | 0.002226 | 222.62 |
| 39 | 2 | 5 | 6 | 1392.8 | 3.2 | 0.002298 | 229.75 |
| 39 | 2 | 5 | 16 | 3105 | 6.1 | 0.001965 | 196.46 |
| 39 | 3 | 15 | 0 | 2970 | 10.9 | 0.00367 | 367 |
| 39 | 2 | 15 | 6 | 1392.8 | 4.9 | 0.003518 | 351.81 |
| 39 | 3 | 25 | 0 | 7281.5 | 32.6 | 0.004477 | 447.71 |
| 46.5 | 1 | 0 | 0 | 14958 | 50 | 0.003343 | 334.27 |
| 46.5 | 2 | 5 | 6 | 1392.8 | 4.9 | 0.003518 | 351.81 |
| 46.5 | 2 | 5 | 16 | 3105 | 9.2 | 0.002963 | 296.3 |
| 46.5 | 2 | 5 | 26 | 1149 | 3.4 | 0.002959 | 295.91 |
| 46.5 | 3 | 15 | 0 | 2970 | 16.3 | 0.005488 | 548.82 |
| 46.5 | 2 | 15 | 6 | 1392.8 | 7.3 | 0.005241 | 524.12 |
| 46.5 | 3 | 25 | 0 | 7281.5 | 49 | 0.006729 | 672.94 |
| 53.5 | 1 | 0 | 0 | 12010 | 66 | 0.005495 | 549.54 |
| 53.5 | 3 | 15 | 0 | 1174 | 11.9 | 0.010136 | 1013.63 |
| 53.5 | 2 | 15 | 16 | 1526.8 | 7.8 | 0.005109 | 510.87 |
| 53.5 | 2 | 15 | 26 | 2335 | 12.9 | 0.005525 | 552.46 |
| 53.5 | 3 | 25 | 0 | 5102.5 | 54.4 | 0.010661 | 1066.14 |
| 53.5 | 2 | 25 | 6 | 906.3 | 7.1 | 0.007834 | 783.41 |
| 53.5 | 2 | 25 | 16 | 1526.8 | 11.9 | 0.007794 | 779.41 |
| 53.5 | 3 | 35 | 0 | 2551.3 | 32.6 | 0.012778 | 1277.78 |
| 53.5 | 2 | 35 | 6 | 906.3 | 10.5 | 0.011586 | 1158.56 |
| 61 | 1 | 0 | 0 | 12010 | 98.9 | 0.008235 | 823.48 |
| 61 | 2 | 15 | 16 | 1526.8 | 11.9 | 0.007794 | 779.41 |
| 61 | 2 | 15 | 26 | 2335 | 19.4 | 0.008308 | 830.84 |
| 61 | 2 | 25 | 6 | 906.3 | 10.5 | 0.011586 | 1158.56 |
| 61 | 2 | 25 | 16 | 1526.8 | 17.7 | 0.011593 | 1159.29 |
| 61 | 3 | 35 | 0 | 2551.3 | 49 | 0.019206 | 1920.59 |
| 61 | 2 | 35 | 6 | 906.3 | 16 | 0.017654 | 1765.42 |
| 61 | 3 | 45 | 0 | 4367 | 125.8 | 0.028807 | 2880.7 |
| 70 | 1 | 0 | 0 | 11466 | 273.7 | 0.023871 | 2387.06 |

| Age (y) | Current Smoking Status[1] | Years Smoked | Years Quit | Person Years | Number of Deaths (All-Cause) | Mortality Rate ($_nm_x$) | MRP |
|---|---|---|---|---|---|---|---|
| 70 | 2 | 25 | 16 | 1274 | 35.4 | 0.027787 | 2778.65 |
| 70 | 2 | 25 | 26 | 3507 | 73.1 | 0.020844 | 2084.4 |
| 70 | 3 | 35 | 0 | 963 | 39.1 | 0.040602 | 4060.23 |
| 70 | 2 | 35 | 6 | 977 | 23.8 | 0.02436 | 2436.03 |
| 70 | 2 | 35 | 16 | 1274 | 53 | 0.041601 | 4160.13 |
| 70 | 3 | 50 | 0 | 3285 | 136 | 0.0414 | 4140.03 |
| 80 | 1 | 0 | 0 | 4486 | 243.6 | 0.054302 | 5430.23 |
| 80 | 3 | 35 | 0 | 138 | 14.4 | 0.104348 | 10434.78 |
| 80 | 2 | 35 | 16 | 335.5 | 19.2 | 0.057228 | 5722.8 |
| 80 | 2 | 35 | 26 | 1442 | 80.4 | 0.055756 | 5575.59 |
| 80 | 3 | 55 | 0 | 740 | 50.4 | 0.068108 | 6810.81 |
| 80 | 2 | 55 | 6 | 253 | 19.2 | 0.075889 | 7588.93 |
| 80 | 2 | 55 | 16 | 335.5 | 28.8 | 0.085842 | 8584.2 |

1 = Never-Tobacco User, 2 = Former Smoker, 3 = Current Smoker

## 2.3. Excess Relative Risks (ERR) Ratios

ERR is the differential amount of risk posed by using one tobacco product (e.g., smokeless tobacco) relative to using another tobacco product (e.g., cigarettes), measured in terms of mortality rates. The mortality models employed in these analyses estimated the mortality rate of current MST users by adjusting the mortality rate of current cigarette smokers using the ERR of current ST users relative to current cigarette smokers.

The Linked Mortality Analysis [6] assessed the relationship between mortality and tobacco use in the form of smokeless tobacco use or cigarette use by linking nationally representative, cross-sectional public health surveys data from NHIS to prospective mortality follow-up data from the National Death Index.

Cox proportional hazards models were used to fit the data and inferences were made on the basis of model-adjusted hazard ratios (HRs). The following covariates were included: age, race, sex, body mass index, education, family income, self-assessed health status, tobacco use, and cigarettes per day. Smokeless tobacco use was defined as snuff use, chewing tobacco use, or both. Pooling snuff and chewing tobacco use is reasonable because the all-cause mortality risks associated with snuff and chewing tobacco use are not different. We assume the all-cause mortality risk of MST to be equal to the mortality risks of ST obtained

from the Linked Mortality Analysis. HRs derived from the Linked Mortality Analysis[1] and calculated ERRs are shown in Table S2.6. We used the HR estimates from the publicly available NHIS dataset because we wanted others in public health to be able to replicate our analysis. The publicly available HR estimates were different from the restricted dataset; however, we believe that this will not impact the model outcome significantly, since we conducted a sensitivity analysis using a wider range of ERR ratios.

**Table S2.6. All-Cause Mortality HRs for Male Survey Respondents: Users of Various Tobacco Products vs. Never-Users of Tobacco Products**

| Tobacco User | Definition | Notation | Hazard Ratio (95%CI) |
|---|---|---|---|
| Never User of Tobacco | Respondents who have never used any tobacco products | $HR_{NT}$ | 1.000 |
| **Inputs for the calculation of the ERR of current MST users compared with current cigarette smokers** | | | |
| Current ST and Never-Smoker | Respondents who are using ST and have never used cigarettes | $HR_{MST}$ | 1.101 (0.954, 1.271) |
| Current Smokers and Never-ST | Respondents who are currently smoking and have never used STs | $HR_{CS}$ | 2.120 (1.977, 2.273) |
| **Inputs for the calculation of the ERR of former MST users compared with former cigarette smokers** | | | |
| Former ST and Never-Smoker | Respondents who have quit ST and have never used cigarettes | $HR_{FMST}$ | 1.010 (0.906, 1.126) |
| Former Smoker and Never-ST | Respondents who have quit smoking and have never used STs | $HR_{FCS}$ | 1.280 (1.202, 1.362) |

CI = confidence interval; CS = current smoker; ERR = excess relative risk; FCS = former cigarette smoker; FMST = former moist smokeless tobacco user; HR = hazard ratio; MST = moist smokeless tobacco; NHIS = National Health Interview Survey; ST = smokeless tobacco.

1 Cox proportional hazards models are limited to respondents who are never-users of tobacco, or respondents who are never-users of pipe tobacco or cigars and who are current or former ST users, current or former smokers.

2 All Cox proportional hazards models control for tobacco use, age, race/ethnicity, body mass index, educational attainment, family income, and self-assessed health status at the time the survey was administered.

3 Results are based on data from NHIS 1987, 1991, 1992, 1998, 2000, and 2005 and reflect only male respondents:
- counts of total records = 58,615 with 10,287 deaths;
- never-users of tobacco = 27,531 with 3,162 deaths;
- current smokers = 15,815 with 3,239 deaths;
- former smokers = 13,091 with 3,669 deaths;
- current ST users = 2,575 with 400 deaths; and

[1] HRs used to derive ERRs are based on analysis of the National Health Interview Survey public use files (survey years: 1987, 1991, 1992, 1998, 2000, 2005) linked to the 2011 National Death Index update.

- former ST users = 3, 463 with 555 deaths.

The ERR ratio for exclusively using MST compared to smoking is derived from relative risk, where:

$$ERR_{MST/CS} = \frac{RR_{MST} - 1}{RR_{CS} - 1}$$

The baseline ERR ratio for never-tobacco users is 0, while the baseline ERR for current cigarette smokers is 1. In this context, we can calculate the ERR ratios of current MST users compared with current cigarette smokers and former MST users compared with former cigarette smokers.

The ERR ratio of current MST users compared with current cigarette smokers is calculated as 0.09:

$$ERR_{MST/CS} = \frac{HR_{MST}-1}{HR_{CS}-1} = \frac{1.101-1}{2.120-1} = 0.09,$$

where the HR values 1.101 and 2.120 are taken from Table S2.6.

The ERR ratio of former MST users compared with former cigarette smokers is 0.04:

$$ERR_{FMST/FCS} = \frac{HR_{FMST}-1}{HR_{FCS}-1} = \frac{1.010-1}{1.280-1} = 0.04,$$

where the HR values 1.101 and 1.280 are taken from Table S2.6.

## References


1. Tam, J.; Day, H.R.; Rostron, B.L.; Apelberg, B.J. A systematic review of transitions between cigarette and smokeless tobacco product use in the United States. *BMC Public Health* **2015**, *15*, 258.
2. Tomar, S.L. Is use of smokeless tobacco a risk factor for cigarette smoking? The U.S. experience. *Nicotine Tob. Res.* **2003**, *5*, 561-569.
3. Wetter, D.W.; McClure, J.B.; de Moor, C.; Cofta-Gunn, L.; Cummings, S.; Cinciripini, P.M.; Gritz, E.R. Concomitant use of cigarettes and smokeless tobacco: prevalence, correlates, and predictors of tobacco cessation. *Prev. Med.* **2002**, *34*, 638-648.
4. Zhu, S.-H.; Wang, J.B.; Hartman, A.; Zhuang, Y.; Gamst, A.; Gibson, J.T.; Gilljam, H.; Galanti, M.R. Quitting cigarettes completely or switching to smokeless tobacco: Do U.S. data replicate the Swedish results? *Tob. Control* **2009**, *18*, 82-87.
5. Friedman, G.D.; Tekawa, I.; Sadler, M.; Sidney, S. Smoking and mortality: the Kaiser Permanente experience. In *Changes in Cigarette-Related Disease Risks and Their Implications for Prevention and Control*, Shopland, D.R., Burns, D.M., Garfinklel, L., Samet, J.M., Eds. U.S. Department of Health and Human Services, National Institutes of Health, National Cancer Institute: Rockville, MD, 1997; pp. 477-499.
6. Fisher, M.T.; Tan-Torres, S.M.; Gaworski, C.L.; Black, R.A.; Sarkar, M. Smokeless tobacco mortality risks: an analysis of two contemporary nationally representative longitudinal mortality studies. *Harm Reduct. J.*, in revision.
