# Supplementary material for: A Computational Model for Assessing the Population Health Impact of Introducing a Modified Risk Claim on an Existing Smokeless Tobacco Product
Source: Int J Environ Res Public Health. 2019 Apr 9;16(7):1264. doi: 10.3390/ijerph16071264 (PMC6479389; doi:10.3390/ijerph16071264)
Supplement: Supplementary file 1 [file ijerph-16-01264-s001.zip › Supplementary File 3- updated_03142019.pdf]

A Computational Model for Assessing the Population Health Impact of Introducing a Modified Risk Claim on an Existing Smokeless Tobacco Product

Raheema S. Muhammad-Kah, Yezdi B. Pithawalla, Edward L. Boone, Lai Wei, Michael A. Jones, Ryan A. Black, Bryan Thomas, Mohamadi A. Sarkar

Supplementary File 3. MODEL VALIDATION

3.1. Single cohort model

This section discusses validation of the ALCS Cohort Model. The goal of the validation process is to compare outcomes from the model with the number of survivors estimated using mortality data reported in U.S. Life Tables by the Centers for Disease Control and Prevention (CDC) [1]. The validation of the Base Case assumes the world as-is today in which both cigarettes and Moist Smokeless Tobacco (MST) are marketed in the U.S., but MST is marketed without any reduced risk claim. As discussed in the publication, in this framework, these two products also coexist in the Modified Case scenario, but reflect authorization for MST to be marketed with a reduced risk claim.

To validate the Base Case model, we compared number of survivors predicted by the model, as a cohort of 1MM males starting at age 13 ages over time, against estimates using mortality data reported in the 2006 U.S. life table from the National Vital Statistics Report [1]. Initiation and cessation rates for the two products (i.e., cigarettes and ST) reflect those in 1980 ([3], [4], and [5])]. The mean, and the percent difference between the model predicted mean and the reported mean were calculated. Table S3.1 shows the validation results, which yielded a low percent difference for all age groups. As expected in term of all- cause mortality we observe a closer agreement between the two estimates in the older ages when tobacco-related mortality is more prevalent.

Table S3.1: Base Case Single-Cohort Model Validation: Comparison of Number of Survivors Predicted by the Model with Reported Estimates from the 2006 U.S. Life Table Report for Males.

Age (y)	Number of Survivors [Estimation from U.S. Life Table 2006[1]]	Number of Survivors [Model Prediction]	%Difference[2]
18	997,059	999,885	0.28%
23	990,519	999,659	0.92%
28	983,192	999,112	1.62%
33	976,111	997,654	2.21%
38	967,974	993,989	2.69%
43	956,428	985,739	3.06%
48	938,961	969,342	3.24%

Age (y)	Number of Survivors [Estimation from U.S. Life Table 2006[1]]	Number of Survivors [Model Prediction]	%Difference[2]
53	912,829	940,850	3.07%
58	876,559	897,629	2.40%
63	826,599	838,642	1.46%
68	757,310	762,409	0.67%
73	663,656	667,201	0.53%

[1] The method used for estimating the number of survivors from the 2006 U.S. Life Table data [1]. Note: In the ALCS Cohort model, survival of an initial cohort of 1,000,000 males is followed in 5-year intervals, starting from age 13 years. Also all possible transitions do not occur until age group 38 years (i.e. the 38- 42 years, 5 year interval).

[2] % Difference is calculated as 100 × [(predicted mean - reported mean) / reported mean].

3.2. Multiple cohort model

To build the multiple-cohort model, each age group cohort must begin at its corresponding birth time interval, which requires that the cohort size correspond with the number of native-born U.S. males ages 0 to 4 years, as reported by the U.S. Census Bureau since 1900 [6]. Figure S3.1 shows a plot of the 0- to 4-year-old population sizes from 1900 to 2010 based on U.S. Census data. Notice that the plot shows the "baby boom" in the 0- to 4-year-old population from 1940 to 1965, before declining afterwards. Table S3.2 shows the development of the age group cohorts from 1910 to 2014. To validate our multiple-cohort approach, the summation of the cohorts across age groups for the 2010-2014 population in Table S3.2 is compared with the estimate of the 2015 U.S. native-born male population reported by the U.S. Census Bureau.

Initiation and cessation rates for each single-cohort model are obtained from Anderson et al. and Tam et al. [2,5]. Anderson et al. reported yearly initiation and cessation rates by age for 5-year birth cohorts of males born between 1910 and 1980. Given that our age groups are in 5-year intervals, initiation and cessation rates were calculated to reflect this same time frame by averaging the yearly values across 5 years. For initiation and cessation rates of males born after 1980, data from Tam et al. were used.

The value of 140,297,313 males projected by the model compares well with the 2015 U.S. Census estimate of 137,187,000 native-born males. The comparison shows that the percent difference between the population generated using our multiple cohort approach and the estimate reported in the U.S. Census data is only 2.27 percent.

Figure S3.1: U.S. Male Population Aged Between 0 and 4 Years from 1910 to 2010 (U.S. Census Bureau)

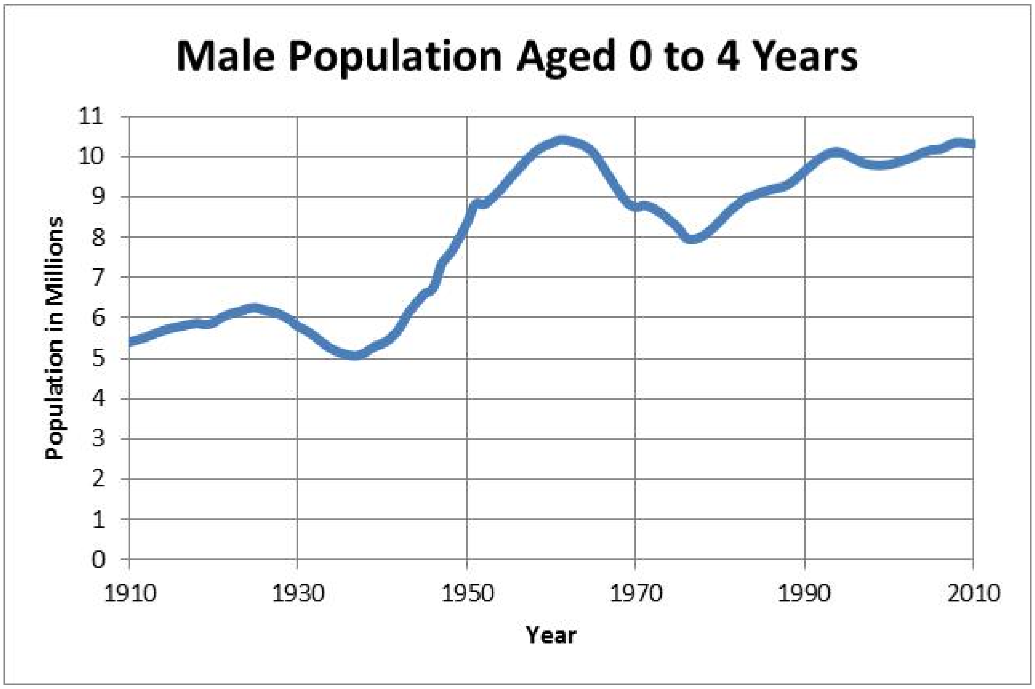

Table S3.2: Cohort Population Sizes by Age Group from 1915 to 2015

	1915	1920	1925	1930	1935	1940	1945	1950	1955	1960	1965	1970	1975	1980	1985	1990	1995	2000	2005	2010	2015
AGE GROUPS	1910-1914	1915-1919	1920-1924	1925-1929	1930-1934	1935-1939	1940-1944	1945-1949	1950-1954	1955-1959	1960-1964	1965-1969	1970-1974	1975-1979	1980-1984	1985-1989	1990-1994	1995-1999	2000-2004	2005-2009	2010-2014
0-4	5,745,000	5,889,000	6,260,000	5,787,000	5,147,600	5,372,700	6,608,900	8,362,000	9,449,100	10,338,800	10,089,800	8,751,100	8,240,000	8,414,200	9,126,900	9,649,600	10,044,500	9,810,600	10,175,600	10,317,900	10,065,000
5-9		5,735,141	5,878,895	6,249,257	5,777,069	5,138,766	5,363,480	6,597,558	8,347,648	9,432,887	10,321,057	10,072,487	8,736,083	8,225,861	8,399,762	9,111,239	9,633,042	10,027,264	9,793,766	10,158,139	10,300,195
10-14			5,723,460	5,866,921	6,236,528	5,765,300	5,128,298	5,352,556	6,584,120	8,330,645	9,413,673	10,300,033	10,051,966	8,718,287	8,209,108	8,382,655	9,092,680	9,613,420	10,006,839	9,773,816	10,137,448
15-19				5,709,327	5,852,433	6,221,128	5,751,063	5,115,636	5,339,338	6,567,863	8,310,071	9,390,427	10,274,598	10,027,145	8,696,756	8,188,834	8,361,954	9,070,226	9,589,680	9,982,128	9,749,680
20-24					5,691,869	5,834,537	6,202,105	5,733,478	5,099,993	5,323,011	6,547,779	8,284,661	9,361,712	10,243,180	9,996,487	8,670,162	8,163,795	8,336,385	9,042,491	9,560,357	9,951,605
25-29						5,664,203	5,805,811	6,172,304	5,705,317	5,075,067	5,297,066	6,516,594	8,246,130	9,318,914	10,197,883	9,952,938	8,632,351	8,127,625	8,300,333	8,994,419	9,509,532
30-34							5,617,985	5,757,784	6,123,859	5,660,020	5,035,426	5,256,438	6,468,727	8,188,029	9,255,875	10,131,849	9,889,870	8,577,449	8,075,488	8,250,766	8,910,368
35-39								5,549,486	5,687,742	6,053,947	5,594,890	4,978,522	5,198,368	6,400,661	8,105,495	9,166,562	10,038,557	9,800,916	8,500,282	8,004,376	8,183,908
40-44									5,453,510	5,589,869	5,956,259	5,503,997	4,899,156	5,117,297	6,305,690	7,990,109	9,041,597	9,907,792	9,676,582	8,395,340	7,907,831
45-49										5,323,523	5,457,490	5,823,544	5,380,626	4,791,242	5,007,003	6,176,060	7,831,874	8,869,569	9,726,992	9,508,959	8,253,932
50-54											5,152,903	5,283,934	5,648,177	5,217,786	4,648,526	4,860,903	6,003,034	7,619,372	8,637,308	9,486,952	9,285,499
55-59												4,940,905	5,068,209	5,428,329	5,013,999	4,469,635	4,677,119	5,783,733	7,348,137	8,336,955	9,173,452
60-64													4,680,653	4,802,969	5,154,974	4,760,999	4,246,739	4,447,008	5,506,304	7,000,690	7,949,850
65-69														4,359,189	4,474,561	4,812,127	4,443,880	3,966,114	4,155,736	5,150,042	6,552,272
70-74															3,954,929	4,060,613	4,374,347	4,039,083	3,606,279	3,779,274	4,686,909
75-79																3,437,255	3,529,573	3,806,388	3,514,026	3,137,564	3,288,464
80-84																	2,776,359	2,850,893	3,074,701	2,838,648	2,534,707
85-89																		1,974,578	2,027,920	2,184,653	2,018,930
90-94																			1,126,028	1,158,578	1,243,008
95-99																				448,060	463,860
100-104																					130,863
																	Total Model Population				140,297,313
																	Projected Native male Population (US Census)				137,187,000
																	Percent Difference				2.27%

Note: cells with the same color correspond to the same cohort.

References

1. Arias, E. United States life tables, 2006. *Natl. Vital Stat. Rep.* **2010**, *58*, 1-40.
2. Anderson, C.M.; Burns, D.M.; Dodd, K.W.; Feuer, E.J. Chapter 2: Birth-cohort-specific estimates of smoking behaviors for the U.S. population. Risk Anal. **2012**, 32, 14-24.
3. Messer, K.; Pierce, J.P.; Zhu, S.H.; Hartman, A.M.; Al-Delaimy, W.K.; Trinidad, D.R.; Gilpin, E.A. The California Tobacco Control Program's effect on adult smokers: (1) Smoking cessation. *Tob. Control* **2007**, *16*, 85-90.
4. Pierce, J.P. International comparisons of trends in cigarette smoking prevalence. *Am. J. Public Health* **1989**, *79*, 152-157.
5. Tam, J.; Day, H.R.; Rostron, B.L.; Apelberg, B.J. A systematic review of transitions between cigarette and smokeless tobacco product use in the United States. *BMC Public Health* **2015**, *15*, 258.
6. U.S. Census Bureau. Population in the United States. **2016**.
